# Designing a novel fractional order mathematical model for COVID-19 incorporating lockdown measures

**DOI:** 10.1038/s41598-023-50889-5

**Published:** 2024-02-05

**Authors:** Waleed Adel, Hatıra Günerhan, Kottakkaran Sooppy Nisar, Praveen Agarwal, A. El-Mesady

**Affiliations:** 1 Department of Mathematics and Engineering Physics, Faculty of Engineering, Mansoura University, Mansoura, 35511 Egypt; 2 Laboratoire Interdisciplinaire de l’Université Française d’Egypte (UFEID Lab), Université Française d’Egypte, Cairo, 11837 Egypt; 3 Department of Mathematics, Faculty of Education, Kafkas University, Kars, Turkey; 4 MEU Research Unit, Middle East University, Amman, Jordan; 5 Department of Mathematics, College of Science and Humanities in Alkharj, Prince Sattam Bin Abdulaziz University, Alkharj, 11942 Saudi Arabia; 6 School of Technology, Woxsen University, Hyderabad, 502345 Telangana India; 7 Department of Mathematics, Anand International College of Engineering, Jaipur, 303012 India; 8 Nonlinear Dynamics Research Center (NDRC), Ajman University, Ajman, United Arab Emirates; 9 International Center for Basic and Applied Sciences, Jaipur, 302029 India; 10 Department of Physics and Engineering Mathematics, Faculty of Electronic Engineering, Menoufia University, Menouf, 32952 Egypt

**Keywords:** Computational biology and bioinformatics, Mathematics and computing

## Abstract

This research focuses on the design of a novel fractional model for simulating the ongoing spread of the coronavirus (COVID-19). The model is composed of multiple categories named susceptible $S(t)$, infected $I(t)$, treated $T(t)$, and recovered $R(t)$ with the susceptible category further divided into two subcategories ${S}_{1} (t)$ and ${S}_{2} (t)$. In light of the need for restrictive measures such as mandatory masks and social distancing to control the virus, the study of the dynamics and spread of the virus is an important topic. In addition, we investigate the positivity of the solution and its boundedness to ensure positive results. Furthermore, equilibrium points for the system are determined, and a stability analysis is conducted. Additionally, this study employs the analytical technique of the Laplace Adomian decomposition method (LADM) to simulate the different compartments of the model, taking into account various scenarios. The Laplace transform is used to convert the nonlinear resulting equations into an equivalent linear form, and the Adomian polynomials are utilized to treat the nonlinear terms. Solving this set of equations yields the solution for the state variables. To further assess the dynamics of the model, numerical simulations are conducted and compared with the results from LADM. Additionally, a comparison with real data from Italy is demonstrated, which shows a perfect agreement between the obtained data using the numerical and Laplace Adomian techniques. The graphical simulation is employed to investigate the effect of fractional-order terms, and an analysis of parameters is done to observe how quickly stabilization can be achieved with or without confinement rules. It is demonstrated that if no confinement rules are applied, it will take longer for stabilization after more people have been affected; however, if strict measures and a low contact rate are implemented, stabilization can be reached sooner.

## Introduction

Many diseases have had devastating consequences for human life over many years and decades. For example, Ebola is one of those deadly diseases that can be transmitted from infected animals, like fruit bats, to uninfected humans. For example, Hepatitis B is one of the viruses that has a deadly effect on infected individuals. It has an original form that has been transmitted over the years from Chimps to humans. This was not noticed with symptoms during the nineteenth century through its transmission. Five countries reported the spread of this disease, and more than 300,000 humans were infected with it. The role of mathematical modeling is crucial in simulating such a disease to better understand the dynamics of this disease, which suppresses the spread of such a virus. With the aid of mathematical modeling, researchers can suggest and assess several intervention techniques that may help slow down the virus. In addition, it plays an important role in estimating key epidemiological parameters such as the basic reproduction number (${R}_{0}$), which represents the average number of new infections caused by a single infected individual. Understanding ${R}_{0}$ aids in predicting the potential for disease spread and designing effective control strategies. Additionally, mathematical modeling can be used to analyze the impact of vaccination programs, antiviral treatment, and behavior change interventions on the prevalence of Hepatitis B. Researchers have been using these models to gain better insight into optimal strategies for prevention, screening, and treatment, guiding public health policies and resource allocation. For example, Din et al.^1,2^ proposed a fractional model based on the definition of Caputo and Atangana-Baleanu Caputo (ABC) derivatives, respectively, to highlight the effect of vaccination and immunization on the population, slowing down the spread of Hepatitis B. In addition, another work by Liu et al.^3^ proposed another fractional model for simulating the Hepatitis B virus model with non-singular and non-local kernels. Moreover, Sabbar et al.^4^ investigated the solution of fractal–fractional differentiation and independent quadratic Lévy jumps on the dynamics of a general epidemiological model. Other examples of deadly diseases that have been simulated using mathematical models may include Lassa fever^5,6^, Influenza virus^7,8^, Monkeypox virus^9–12^, Zika virus^13^, Leptospirosis virus^14^ and Lassa disease^15^. All of these viruses have a life-threatening effect on human lives. Therefore, the need to create several medical operations and measures to stop these diseases and others like them from spreading, along with an effective cure, is a must.

By the end of 2019, the new Middle East respiratory syndrome (COVID-19) emerged in China, specifically from the seafood markets in Wuhan, and since then it has been spreading to all parts of the world. Both undeveloped and developing countries have been reporting new cases daily, leading to an alarming increase in infections. The total number of confirmed cases until now is more than 676 million individuals, with a total death rate of more than 6 million, according to the WHO^16^. The virus is identified spreading through small air sole parts ejaculated from the infected person while sneezing or coughing to another uninfected person, causing respiratory infection, and is considered highly infectious when the infected person coughs or sneezes^17^. The droplets are considered heavy and may land on surfaces near the infected person after coughing, causing the infection in some uninfected populations when touching the contaminated surface with their eye, mouth, or nose, which may cause them to catch the virus. The symptoms of the virus can range from mild to gradually worsening, with nearly every infected individual experiencing them. One out of every six patients may require hospital admission for respiratory difficulties, and elderly adults with underlying medical illnesses are particularly affected. To slow the spread of COVID-19, lockdowns, mandating masks in public places, and promoting social distancing are some of the effective methods that countries have taken to ensure a slow rate of infection. These measures have helped to some degree in containing the spread of the virus.

The signs of the infection COVID-19 may vary from person to person, but the most common are a fever of 38 °C or higher, a dry cough, and muscle pain. Other less frequent symptoms may include a sore throat, among others. These symptoms usually start mild and become more severe over time. Most of the infected individuals experience symptoms that can be treated without special medications; however, some medications may be used to reduce the severity of the symptoms. In rare cases, approximately one in six people becomes severely ill and requires hospitalization due to difficulty breathing. To better understand the complexity of the virus and its effect on human health, several factors have to be taken into consideration. It should be noted that the true cause of the illness remains unknown, although there is a potential connection to wild animals, such as bats. The incubation period for the virus ranges from 2 to 14 days, depending on the health condition of the infected individual and the treatment he can get. The primary goal of any treatment is to boost the patient's immune system to better fight off the infection. Until the body can fight off the infection, different types of medications have been used to treat the symptoms of the infection. This was the only solution until a new vaccine was distributed in 2022. Several vaccines have been developed, including Modena, Pfizer, and Johnson & Johnson. These vaccines have been helping to slow the spread of the virus, making it easier for mankind to get back to their normal lives. Until today, more than 13 billion doses of vaccines have been administered to the world population, helping a huge part of the world get back to a normal life. Still, the threat of the emergence of new variants of COVID-19 can be predicted using mathematical simulation, especially the fractional order models.

Fractional differential equations (FDEs) have been playing a major role in understating the dynamics of real-life phenomena. Not only in understanding the complex dynamics of biological systems, but it has also been used in other fields such as thermoelectricity. The use of FDEs has been increasing throughout the last few years, making them one of the most important tools to be used in simulations. There have been several definitions of fractional operators, each of which has advantages and drawbacks. One of the most important and original definitions is the Caputo fractional derivative^18^. This was introduced by Michel Caputo in the 1960s as a way to generalize the classical derivative and provide a framework for modeling and analyzing phenomena that exhibit fractional-order dynamics. The Caputo fractional derivative possesses several advantages over other definitions. One key advantage is its ability to handle the initial conditions of any problem that involves a fractional derivative. In addition, unlike other definitions, the Caputo fractional derivative allows for the inclusion of initial conditions involving integer-order derivatives. This makes it useful in modeling real-world processes that involve fractional-order dynamics and have initial conditions with integer-order derivatives. Moreover, it allows for the formulation of fractional differential equations that capture the memory and hereditary properties of systems, enabling a more accurat e representation of their dynamics. This is especially valuable in modeling systems with long-term memory effects or systems exhibiting anomalous diffusion and power-law behavior. Several researchers have been using this definition to understand the complex behavior and dynamics of the spread of the COVID-19 pandemic. For example, Khan and Atangana in Ref.^19^ first modeled a fractional model and described its dynamics. Alkahtani et al.^20^ proposed a numerical approach for solving a fractional-order model with the aid of Lagrange polynomials. The spreading of the virus in Indonesia has been investigated, and the stability analysis of the model has been examined. Moreover, Sabir et al.^21^ applied a heuristic computational technique for simulating the behavior of the SITR COVID-19 model with the aid of the morlet wavelet neural network. Okuonghae et al.^22^ employed a mathematical model for understanding the spreading behavior of COVID-19 with real-life data from Nigeria. To help in controlling the virus, Djaoue et al.^23^ proposed a model that takes into account the transmission and mitigation of control strategies with data from Cameroon. Other models can be found in Ref.^24–28^ and references therein. These models, among others, participated in controlling the spread of the virus, which reached the world in a safer environment.

In this research, we are also exploring the potential of using the Laplace Adomian decomposition method (LADM) to solve the fractional COVID-19 model. This method is a powerful yet straightforward approach to tackling epidemic models and has been successfully applied in biology, engineering, and applied mathematics. It combines the Laplace transform and the Adomian decomposition method, offering several advantages for solving complex problems. One of the advantages of this method is its accuracy, as by employing the Laplace transform, it transforms the differential equations into algebraic equations, which are often easier to solve. This transformation reduces the complexity of the problem and enables the use of powerful algebraic techniques to obtain accurate solutions. Additionally, the Adomian decomposition method provides a systematic and robust approach to handling nonlinear terms, allowing for accurate approximation of the solution even in the presence of nonlinearity. This method does not require any perturbation or linearization, nor does it need a defined size of the step like the Rung-Kutta of order 4 technique. Additionally, it is independent of any parameters, unlike the Homotopy Perturbation Method (HPM), which depends on certain parameters. This has led to its use in solving various models, such as the HIV CD4 + T cell problem^29^, Volterra integrodifferential equations^30^, fractional-order smoking model^31^, epidemic childhood disease model^32^, vector-borne disease model^33^, fractional partial differential equation^34^, RadhakrishnanKunduLakshmanan equation^35^, and other similar and related models.

We are interested in this paper to capture the dynamics of the fractional COVID-19 model, taking into account the effect of the lockdown that several countries have been taking to control the spread of the virus. To the best of our knowledge, this is the first time this model has been solved using the Caputo definition. The novelty of the paper lies in the following points:

1. A novel Caputo fractional COVID-19 has been propped to capture the dynamics of the model, incorporating lockdown measures.
2. The existence, uniqueness, and positivity of the new proposed fractional model are examined in detail, which proves that the presented model has a unique solution.
3. A detailed stability analysis for the model is presented to highlight the stability region and conditions for the model.
4. The results for simulating the different compartments of the model are obtained for different values of the fractional order, and real data from Italy is presented.
5. The results prove that the control measures represented in this case by lockdown have an effect on slowing down the spread of the virus and ending the pandemic.

The organization of the rest of the paper is as follows: The formulation of the model is detailed in Sect. “Model formulation” with the interaction of the different compartments. Section “Basic definitions” provides some basic definitions and fundamentals. Positivity, boundedness, existence, and uniqueness are discussed in detail in Sect. “Positivity, boundedness, existence, and uniqueness”. The stability analysis and the equilibrium points are illustrated in Sect. “Equilibrium points and stability analysis”. The proposed technique for solving the main model is highlighted in Sect. “Proposed technique”, along with a verification with real data. Section “Numerical Simulations” presents the numerical results of the work using different techniques, and the conclusion for the work is provided in Sect. “Conclusion”.

## Model formulation

In this section, we will present the novel COVID-19 model, which is composed of four primary components: susceptible $S$, infected $I$, treated $T$, and recovered $R$. In addition, the susceptible category is divided into two more subcategories: ${S}_{1} (t)$ those that include uninfected individuals and ${S}_{2} (t)$ those that refer to those who are uninfected but have pre-existing health conditions or are elderly. The parameter $I(t)$ is considered the infected person with the virus at the time $t$ and $T\left(t\right)$ is part of the treatment of the virus where no treatment or vaccinations are yet, but some measures can be taken to act against it. With these precautionary measures, the infected person who has recovered from this disease can take the parameter $R(t)$ at a time $t$.

The general form of the fractional SITR model can take the following form^36^,

1 $$\left\{\begin{array}{l}{D}_{*}^{\alpha }{S}_{1}\left(t\right)=\Theta -\beta {S}_{1}\left(t\right)I\left(t\right)-{\zeta }_{1}{S}_{1}\left(t\right)R\left(t\right)-{\gamma S}_{1}\left(t\right)+{\delta }_{1}I\left(t\right)+{\delta }_{2}T\left(t\right)+{\eta }_{1}{S}_{2}\left(t\right),\\ {D}_{*}^{\alpha }{S}_{2}\left(t\right)={\zeta }_{1}{S}_{1}\left(t\right)R\left(t\right)-{\gamma S}_{2}\left(t\right)-{\eta }_{1}{S}_{2}\left(t\right),\\ {D}_{*}^{\alpha }I\left(t\right)=\beta {S}_{1}\left(t\right)I\left(t\right)-{\delta }_{1}I\left(t\right)-{\xi }_{1}I\left(t\right)-\gamma I\left(t\right)-{\zeta }_{2}I\left(t\right)R\left(t\right)+{\eta }_{2}T\left(t\right),\\ {D}_{*}^{\alpha }T\left(t\right)={\zeta }_{2}I\left(t\right)R\left(t\right)-\gamma T\left(t\right)-{\eta }_{2}T\left(t\right)-{\delta }_{2}T\left(t\right)-{\xi }_{2}T\left(t\right),\\ {D}_{*}^{\alpha }R\left(t\right)=\vartheta I\left(t\right)-\psi R\left(t\right),\end{array}\right.$$

With the following conditions,

2 $${S}_{1}\left(0\right)={H}_{1}, {S}_{2}\left(0\right) ={H}_{2}, I\left(0\right)={H}_{3}, T\left(0\right)={H}_{4}, R\left(0\right)={H}_{5}.$$

**Figure 1:**
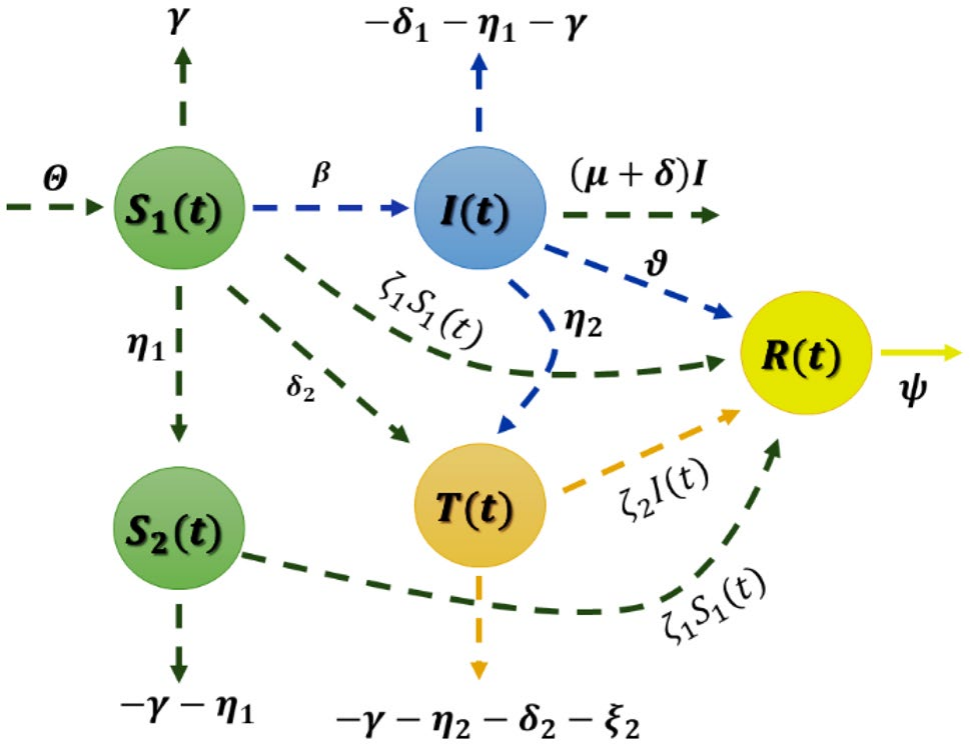
Schematic diagram of the interaction of different compartments.

The order $\alpha$ defines the fractional order in the main model, which can take several values. The above model represents a fractional model of the SITR model with fractal parameters, and the state variable parameters are summarized in Table 1. In addition, Fig. 1 demonstrates the interaction between different compartments of the model.

**Table 1:** State variable definitions for the SITR model.

Parameters	Description
${S}_{1}(t)$	Population of susceptible individuals that aren’t yet under lockdown
${S}_{2}(t)$	Susceptible populations that are under lockdown
$I(t)$	An infected population that isn’t under lockdown
$T(t)$	Infective populations that are under lockdown
$R(t)$	Recovered population after infection
$\Theta$	Recruitment rate
$\beta$	Infection contact rate
${\delta }_{1}$	The recovery rate for infected individuals
${\delta }_{2}$	The recovery rate for treated individuals
${\zeta }_{1}$	Imposing lockdown measures for susceptible individuals
${\zeta }_{2}$	Imposing lockdown measures for infected individuals
${\xi }_{1}$	The death rate for infected individuals
${\xi }_{2}$	Death rate for treated individuals
$\gamma$	Death rate of natural circumstances
${\eta }_{1}$	Transfer rate from susceptible individuals from lockdown to normal class
${\eta }_{2}$	Transfer rate from infected individuals from lockdown to normal class
$\nu$	Lockdown application rate
$\psi$	Lockdown depletion rate

In the next section, we will provide some basic definitions that will be needed later.

## Basic definitions

In this section, some basic definitions will be presented.

### Definition 3.1

Reference^20^ *A real function *$f\left(x\right), x>0$* belongs to the space of *${C}_{\mu }, \mu \epsilon R$* if there is a number* $P>\mu$ *under the condition that *$f\left(x\right)={x}^{p}{f}_{1}\left(x\right)$* where *${f}_{1}\left(x\right) \in C\left[\left.0,\infty \right)\right.$* and *${C}_{\mu }<{C}_{\beta }$* if *$\mu <\beta$*.*

### Definition 3.2

Reference^20^ *A function *$f\left(x\right), x>0$* belongs to the space *${C}_{\mu }^{m}$*, *$m \in N\bigcup \left\{0\right\}$* if* ${f}^{\left(m\right)}{\in C}_{\mu }$.

### Definition 3.3

Reference^37^ *The fractional integral operator of Riemann–Liouville with order *$\alpha >0$* for *$f{\in C}_{\mu }, \mu \ge -1$* can be defined as follows*,

3 $$({J}_{a}^{\alpha }f)(x)=\frac{1}{\Gamma \left(\alpha \right)}{\int }_{a}^{x}(x-\tau {)}^{\alpha -1}f(\tau )d\tau ,x >a,$$

4 $$({J}_{a}^{0}f)(x)=f(x).$$

In addition, the following properties hold, for $f\in {C}_{\mu }, \mu \ge -1, \alpha ,\beta \ge 0,$ and $\gamma >-1$ we have,

5 $$({J}_{a}^{\alpha }{J}_{a}^{\beta }f)(x)=({J}_{a}^{\alpha +\beta }f)(x),$$

6 $$({J}_{a}^{\alpha }{J}_{a}^{\beta }f)(x)=({J}_{a}^{\beta }{J}_{a}^{\alpha }f)(x)$$

7 $${J}_{a}^{\alpha }{x}^{\gamma }=\frac{\Gamma (\gamma +1)}{\Gamma (\alpha +\gamma +1)}{x}^{\alpha +\gamma }.$$

The definition of the fractional order in terms of Riemann-Louville possesses certain advantages when simulating real-world models. To this end, Caputo proposed a better version of ${D}^{\alpha }$ in his work on viscoelasticity^38^, which is summarized below.

### Definition 3.4

Reference^18^ *The Caputo fractional derivative of the function *$f\left(x\right)$* is in the form*,

8 $$\left(D\begin{array}{c}\alpha \\ a\end{array}f\right)\left(x\right)=\left(J\begin{array}{c}m-\alpha \\ a\end{array}{D}^{m}f\right)\left(x\right)=\frac{1}{\Gamma \left(m-a\right)}\underset{a}{\overset{x}{\int }}{{\left(x-t\right)}^{m-\alpha -1}f}^{\left(m\right)}\left(t\right)dt$$

for $$m-1<\alpha <m$$*, *$$m\in N$$*,* $$x>0$$.

### Lemma 3.1

*If *$-1<\alpha <m$* ,* $m\in {\text{N}}$ *and* $\mu \ge -1$, *then,*

9 $$\left({J}_{a}^{\alpha }{D}_{a}^{\alpha }f\right)\left(x\right)=f\left(x\right)-\sum_{k=0}^{m-1}{f}^{k}\left(a\right)\left(\frac{(x-a{)}^{k}}{k!}\right),a\ge 0$$

10 $$({D}_{a}^{\alpha }{J}_{a}^{\alpha }f)(x)=f(x)$$

### Definition 3.5

Reference^30^ *Suppose* $G(s)$ *is the Laplace transform of *$g\left(t\right)$*. Then, the Laplace transform of the Caputo fractional derivative is defined as*,

$${\{}_{0}^{C}{D}_{t}^{\alpha }g(t)\}={s}^{-\alpha }G(s)- \sum_{i=1}^{m-1}{s}^{\alpha -i-1}{g}^{\left(i\right)}\left(0\right),\left(m-1<\alpha \le m\right);m\in {\mathbb{N}}.$$

### Definition 3.6

*For *$t\in {\mathbb{R}}$*, the generalized Mittag–Leffler function *${E}_{p,q}(t)$* is defined by *${E}_{p,q}\left(t\right)=\sum_{m=0}^{\infty }\frac{t}{\Gamma (pm+q)}, p>0,q>0,$* and verifies the following property*^39^:

11 $${E}_{p,q}\left(t\right)=t{E}_{p,p+q}\left(t\right)+\frac{1}{\Gamma (q)}.$$

Then, the Laplace transform of ${t}^{q-1}{E}_{p,q}\left(\pm \lambda {t}^{p}\right)$ is defined by,

12 $$\left\{{t}^{q-1}{E}_{p,q}\left(\pm \lambda {t}^{p}\right)\right\}=\frac{{s}^{p-q}}{{s}^{p}\mp \lambda }.$$

In the next section, we shall provide details on the positivity of the acquired solution along with its boundedness for model (1).

## Positivity, boundedness, existence, and uniqueness

### Positivity and boundedness

In this section, we will provide a detailed study of the positivity and boundedness of the main model (1). We first follow the generalized mean values theorem in Ref.^40^, and we prove that the solution to model (1) is nonnegative and bounded for all time $t\ge {t}_{0}$, where all the parameters and initial conditions have positive values. We need the following lemma.

#### Lemma 4.1.1

*Suppose *$g(t)$* and* ${}_{{t}_{0}}^{C}{D}_{t}^{\alpha }g(t)$ *belong to* $C\left[{t}_{0},{t}_{f}\right].$ *Therefore, we get*,

$$g\left(t\right)=g\left({t}_{0}\right)+\frac{1}{\Gamma \left(\alpha \right)}{}_{{t}_{0}}^{C}{D}_{\varepsilon }^{\alpha }g\left(\varepsilon \right).{\left(t-{t}_{0}\right)}^{\alpha }, {t}_{0}\le \varepsilon \le t, \forall t\in \left({t}_{0},{t}_{f}\right].$$

#### Corollary 4.1.1

*Suppose *$g\left(t\right),$ ${}_{{t}_{0}}^{C}{D}_{t}^{\alpha }g(t)$ *belong* *to *$C\left[{t}_{0},{t}_{f}\right]$, *and* $\alpha \in \left(\mathrm{0,1}\right].$ *From *Lemma 4.1.1* if*,

(i) ${}_{{t}_{0}}^{C}{D}_{t}^{\alpha }g(t)\ge 0,$ $\forall t\in ({t}_{0},{t}_{f})$ *then *$g(t)$* is non-decreasing* $\forall t\in \left[{t}_{0},{t}_{f}\right].$
(ii) ${}_{{t}_{0}}^{C}{D}_{t}^{\alpha }g(t)\le 0,$ $\forall t\in ({t}_{0},{t}_{f})$ *then *$g(t)$* is non-increasing* $\forall t\in \left[{t}_{0},{t}_{f}\right].$

We can now prove the following theorems.

#### Theorem 4.1.1

*The region* ${\Delta }_{+}=\{\left({S}_{1},{S}_{2},I,T,R\right);{S}_{1}>0,{S}_{2}\ge 0,I\ge 0,T\ge 0,R\ge 0\}$ *is a positive invariant for model* (1)*.*

#### Proof

The first step is to prove that model (1) has a unique solution on the period $(0,\infty )$, see Refs.^41,42^. From model (1), we have,

13 $$\left\{\begin{array}{l}{\left.{}_{0}^{C}{D}_{t}^{\alpha }{S}_{1}\right|}_{{S}_{1}=0}=\Theta +{\delta }_{1}I\left(t\right)+{\delta }_{2}T\left(t\right)+{\eta }_{1}{S}_{2}\left(t\right)>0,\\ {\left.{}_{0}^{C}{D}_{t}^{\alpha }{S}_{2}\right|}_{{S}_{2}=0}={\zeta }_{1}{S}_{1}\left(t\right)R\left(t\right)\ge 0,\\ {\left.{}_{0}^{C}{D}_{t}^{\alpha }I\right|}_{I=0}={\eta }_{2}T\left(t\right)\ge 0,\\ {\left.{}_{0}^{C}{D}_{t}^{\alpha }T\right|}_{T=0}={\zeta }_{2}I\left(t\right)R\left(t\right)\ge 0,\\ {\left.{}_{0}^{C}{D}_{t}^{\alpha }R\right|}_{R=0}=\vartheta I\left(t\right)\ge 0.\end{array}\right.$$

Hence the region ${\Delta }_{+}$ is a positive invariant based on Corollary 4.1.1, and the solution of model (1) will remain inside ${\Delta }_{+}$. ∎

We then need the following theorem.

#### Theorem 4.1.2

*The total population for the model* (1) *verifies* $0<N\left(t\right)\le \frac{\Theta }{\gamma }.$

#### Proof

Summing the first four equations of the main mode leads to the following.

14 $${}_{0}^{C}{D}_{t}^{\alpha }N\left(t\right)=\Theta -{\gamma [S}_{1}\left(t\right)+{S}_{2}\left(t\right)+I\left(t\right)+T\left(t\right)]-{\xi }_{1}I\left(t\right)-{\xi }_{2}T\left(t\right),$$

that can be rewritten as follows:

15 $${}_{0}^{C}{D}_{t}^{\alpha }N\left(t\right)\le \Theta -\gamma N\left(t\right),$$

Taking Laplace to transform the defined form Eq. (12) for both sides of Eq. (15) we get,

$${s}^{\alpha }\overline{N }\left(s\right)-{s}^{\alpha -1}N\left(0\right)\le \frac{\Theta }{s}-\gamma \overline{N }\left(s\right),$$

Hence,

$$\overline{N }\left(s\right)\le \frac{{s}^{-1}}{{s}^{\alpha }+\gamma }\Theta +\frac{{s}^{\alpha -1}}{{s}^{\alpha }+\gamma }N\left(0\right).$$

From (11) and (12) and if $\left({S}_{1}\left(0\right),{S}_{2}\left(0\right),I\left(0\right),T\left(0\right)\right)\in\Delta ,$ then,

$$N\left(t\right)\le \Theta {t}^{\alpha }{E}_{\alpha ,\alpha +1}\left(-\gamma {t}^{\alpha }\right)+{E}_{\alpha ,1}\left(-\gamma {t}^{\alpha }\right)N\left(0\right),$$

$$N\left(t\right)\le \Theta {t}^{\alpha }{E}_{\alpha ,\alpha +1}\left(-\gamma {t}^{\alpha }\right)+{E}_{\alpha ,1}\left(-\gamma {t}^{\alpha }\right)=\frac{\Theta }{\gamma }\left[\gamma {t}^{\alpha }{E}_{\alpha ,\alpha +1}\left(-\gamma {t}^{\alpha }\right)+{E}_{\alpha ,1}\left(-\gamma {t}^{\alpha }\right)\right]$$

$$=\frac{\Theta }{\gamma }\left[-\left({E}_{\alpha ,1}\left(-\gamma {t}^{\alpha }\right)-\frac{1}{\Gamma \left(1\right)}\right)+{E}_{\alpha ,1}\left(-\gamma {t}^{\alpha }\right)\right],$$

Hence, we conclude that

16 $$N\left(t\right)\le \frac{\Theta }{\gamma }.$$

From inequality (16) and Theorem 4.1.1, we deduce that $0<N\left(t\right)\le \frac{\Theta }{\gamma }.$∎

### Existence and uniqueness

This subsection is devoted to proving the existence and boundedness of the solution of model (1). We start with the next theorem.

#### Theorem 4.2.1

*There exists a unique solution for the model* (1) *for each non-negative initial condition.*

#### Proof

Let the region $\mathrm{\mho }\times \left(0,T\right]$ be defined in the form,

$$\mathrm{\mho }=\left\{\left({S}_{1},{S}_{2},I,T,R\right)\in {\mathbb{R}}^{5}: {\text{max}}(\left|{S}_{1}\right|,\left|{S}_{2}\right|,\left|I\right|,\left|T\right|,\left|R\right|)\le \mu \right\}.$$

Also, let $A\left(X\right)$ be a mapping where,

$$A\left(Y\right)=\left({A}_{1}\left(Y\right),{A}_{2}\left(Y\right),{A}_{3}\left(Y\right),{A}_{4}\left(Y\right),{A}_{5}\left(Y\right)\right);$$

$${A}_{1}\left(Y\right)=\Theta -\beta {S}_{1}\left(t\right)I\left(t\right)-{\zeta }_{1}{S}_{1}\left(t\right)R\left(t\right)-{\gamma S}_{1}\left(t\right)+{\delta }_{1}I\left(t\right)+{\delta }_{2}T\left(t\right)+{\eta }_{1}{S}_{2}\left(t\right),$$

$${A}_{2}\left(Y\right)={\zeta }_{1}{S}_{1}\left(t\right)R\left(t\right)-{\gamma S}_{2}\left(t\right)-{\eta }_{1}{S}_{2}\left(t\right),$$

$${A}_{3}\left(Y\right)=\beta {S}_{1}\left(t\right)I\left(t\right)-{\delta }_{1}I\left(t\right)-{\xi }_{1}I\left(t\right)-\gamma I\left(t\right)-{\zeta }_{2}I\left(t\right)R\left(t\right)+{\eta }_{2}T\left(t\right),$$

$${A}_{4}\left(Y\right)={\zeta }_{2}I\left(t\right)R\left(t\right)-\gamma T\left(t\right)-{\eta }_{2}T\left(t\right)-{\delta }_{2}T\left(t\right)-{\xi }_{2}T\left(t\right),$$

17 $${A}_{5}\left(Y\right)=\vartheta I\left(t\right)-\psi R\left(t\right).$$

Next, for any $Y,\overline{Y}\in \mathrm{\mho }$, it follows from (17) that,

$$\Vert A\left(Y\right)-A\left(\overline{Y }\right)\Vert =\left|{A}_{1}\left(Y\right)-{A}_{1}\left(\overline{Y }\right)\right|+\left|{A}_{2}\left(Y\right)-{A}_{2}\left(\overline{Y }\right)\right|+\left|{A}_{3}\left(Y\right)-{A}_{3}\left(\overline{Y }\right)\right|+\left|{A}_{4}\left(Y\right)-{A}_{4}\left(\overline{Y }\right)\right|+\left|{A}_{5}\left(Y\right)-{A}_{5}\left(\overline{Y }\right)\right|\le \left(2\beta \mu +{2\zeta }_{1}\mu + \gamma \right)\left|{S}_{1}-{\overline{S} }_{1}\right|+\left({\eta }_{1}+\gamma +{\eta }_{1}\right)\left|{S}_{2}-{\overline{S} }_{2}\right|+\left(2\beta \mu +{2\delta }_{1}+{{2\zeta }_{2}\mu +\xi }_{1}+\gamma +\vartheta \right)\left|I-\overline{I }\right|+\left(2{\delta }_{2}+{2\eta }_{2}+\gamma +{\xi }_{2}\right)\left|T-\overline{T }\right|+\left({2\zeta }_{1}\mu +2{\zeta }_{2}\mu +\psi \right)\left|R-\overline{R }\right|\le G\Vert Y-\overline{Y }\Vert .$$

Then we have,

$$G={\text{max}}\{\left(2\mu (\beta +{\zeta }_{1})+ \gamma \right),\left({\eta }_{1}+\gamma +{\eta }_{1}\right),\left(2(\mu \left(\beta +{\zeta }_{2}\right)+{\delta }_{1}){+\xi }_{1}+\gamma +\vartheta \right),\left(2({\delta }_{2}+{\eta }_{2})+\gamma +{\xi }_{2}\right),\left({2\mu (\zeta }_{1}+{\zeta }_{2})+\psi \right)\}.$$

Thus, the Lipschitz condition is verified for $A\left(Y\right).$ Consequently, model (1) possesses a unique and bounded solution. ∎

The following section will be devoted to determining the system’s equilibrium points and their stability behavior.

## Equilibrium points and stability analysis

In this section, we acquire the equilibrium points for model (1) along with the initial conditions in (2). These points are found by equating the system (1) to zero. This shall result in different types of equilibrium points. The first is defined as the disease-free equilibrium point (DFE) which can take the form of ${E}_{0}=(\frac{\Theta }{\gamma },\mathrm{0,0},\mathrm{0,0}).$ For the DFE, we can find the basic reproduction number ${\mathcal{R}}_{0}$ based on the next-generation matrix technique which can take the following form

18 $${\mathcal{R}}_{0}=\rho \left(A{B}^{-1}\right),$$

where $\rho$ is the spectral radius for $A{B}^{-1}$ and $A,B$ are defined as

$$A=\left[\begin{array}{cc}\frac{\beta \Theta }{\gamma }& 0\\ 0& 0\end{array}\right], B=\left[\begin{array}{cc}\gamma +{\delta }_{1}+{\xi }_{1}& -{\eta }_{2}\\ 0& \gamma +{\delta }_{2}+{\eta }_{2}+{\xi }_{2}\end{array}\right],$$

and hence

$$A{B}^{-1}=\left[\begin{array}{cc}\frac{\beta \Theta }{\gamma (\gamma +{\delta }_{1}+{\xi }_{1})}& \frac{\beta \Theta {\eta }_{2}}{\gamma (\gamma +{\delta }_{1}+{\xi }_{1})(\gamma +{\delta }_{2}+{\eta }_{2}+{\xi }_{2})}\\ 0& 0\end{array}\right]$$

Finally, ${\mathcal{R}}_{0}$ can be represented by

19 $${\mathcal{R}}_{0}=\frac{\beta \Theta }{\gamma (\gamma +{\delta }_{1}+{\xi }_{1})}.$$

**Figure 2:**
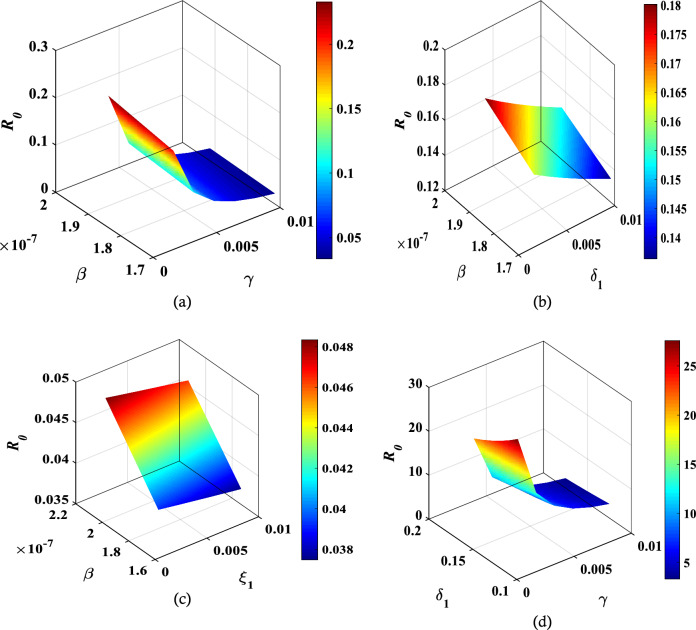
Behaviour of reproduction number ${\mathcal{R}}_{0}$ with different variables.

As can be seen from Eq. (19), the reproduction number ${\mathcal{R}}_{0}$ for the DFE depends on several parameters. The behavior of ${\mathcal{R}}_{0}$ while changing the values of these parameters are illustrated in Fig. 2, where a surface plot illustrates the behavior with different values of $\gamma , {\delta }_{1}, \beta$ and ${\xi }_{1}$.

The second point is the endemic point of EEP. In this case, we have multiple endemic points where the absence of lockdown and measures indicated by ${E}_{1}=\left(\frac{\gamma +{\delta }_{1}+{\xi }_{1}}{\beta },0,\frac{-\gamma +\frac{\beta \theta -\gamma {\delta }_{1}}{\gamma +{\xi }_{1}}}{\beta },\mathrm{0,0}\right)$ and with the presence of the lockdown indicated by ${E}_{2}={(S}_{1}^{*},{S}_{2}^{*},{I}^{*},{T}^{*},{R}^{*}$), where each of the compartments is defined as,

20 $$\left\{\begin{array}{l}{S}_{1}^{*}=\frac{\left(\gamma +{\delta }_{1}+{\xi }_{1}\right)+\frac{\vartheta {\zeta }_{2}\left(\gamma +{\delta }_{2}+{\xi }_{2}\right)}{\psi \left(\gamma +{\delta }_{2}+{\eta }_{2}+{\xi }_{2}\right)}{I}^{*}}{\beta },\\ {S}_{2}^{*}=\frac{\vartheta {\zeta }_{1}\left(\left(\gamma +{\delta }_{1}+{\xi }_{1}\right){I}^{*}+\frac{\vartheta {\zeta }_{2}\left(\gamma +{\delta }_{2}+{\xi }_{2}\right)}{\psi \left(\gamma +{\delta }_{2}+{\eta }_{2}+{\xi }_{2}\right)}{{I}^{*}}^{2}\right)}{\beta \psi \left(\gamma +{\eta }_{1}\right)}\\ {T}^{*}=\frac{\vartheta {\zeta }_{2}}{\psi \left(\gamma +{\delta }_{2}+{\eta }_{2}+{\xi }_{2}\right)}{{I}^{*}}^{2},\\ {R}^{*}=\frac{\vartheta }{\psi }{I}^{*},\end{array},\right.$$

and ${I}^{*}$ can be found by solving the following equation.

$$(\beta \vartheta \psi {\zeta }_{2}(\gamma +{\eta }_{1})(\gamma +{\xi }_{2})+\gamma {\vartheta }^{2}{\zeta }_{1}{\zeta }_{2}(\gamma +{\delta }_{2}+{\xi }_{2})){{I}^{*}}^{2}+(\mathrm{\gamma \vartheta }\psi {\delta }_{1}{\zeta }_{1}+\gamma \vartheta \psi {\delta }_{2}{\zeta }_{2}(\gamma +{\eta }_{1})+\gamma \vartheta \psi {\zeta }_{2}(\gamma +{\eta }_{1})(\gamma +{\xi }_{2})+\beta \gamma {\psi }^{2}(\gamma +{\eta }_{1})(\gamma +{\delta }_{2}+{\eta }_{2}+{\xi }_{2})+\beta {\psi }^{2}(\gamma +{\eta }_{1}){\xi }_{1}(\gamma +{\delta }_{2}+{\eta }_{2}+{\xi }_{2})+\gamma \vartheta \psi {\zeta }_{1}(\gamma +{\xi }_{1})(\gamma +{\delta }_{2}+{\eta }_{2}+{\xi }_{2})){I}^{*}+({\gamma }^{2}-\beta \theta ){\psi }^{2}(\gamma +{\eta }_{1})(\gamma +{\delta }_{2}+{\eta }_{2}+{\xi }_{2})+\gamma {\psi }^{2}{\delta }_{1}(\gamma +{\eta }_{1})(\gamma +{\delta }_{2}+{\eta }_{2}+{\xi }_{2})+\gamma {\psi }^{2}(\gamma +{\eta }_{1}){\xi }_{1}(\gamma +{\delta }_{2}+{\eta }_{2}+{\xi }_{2})=0.$$

The Jacobian matrix for model (1) can be obtained in the following form,

21 $$J=\left(\begin{array}{ccccc}-\beta I-\gamma -{\zeta }_{1}R& {\eta }_{1}& -\beta {S}_{1}+{\delta }_{1}& {\delta }_{2}& -{\zeta }_{1}{S}_{1}\\ {\zeta }_{1}R& -\gamma -{\eta }_{1}& 0& 0& {\zeta }_{1}{S}_{1}\\ \beta I& 0& \beta {S}_{1}-\gamma -{\delta }_{1}-{\zeta }_{2}R-{\xi }_{1}& {\eta }_{2}& -{\zeta }_{2}I\\ 0& 0& {\zeta }_{2}R& -\gamma -{\delta }_{2}-{\eta }_{2}-{\xi }_{2}& {\zeta }_{2}I\\ 0& 0& \vartheta & 0& -\psi \end{array}\right).$$

For the DFE, the Jacobian matrix at ${E}_{0}$ is given by

$${J}_{{E}_{0}}=\left(\begin{array}{ccccc}-\gamma & {\eta }_{1}& -\frac{\beta \theta }{\gamma }+{\delta }_{1}& {\delta }_{2}& -\frac{\theta {\zeta }_{1}}{\gamma }\\ 0& -\gamma -{\eta }_{1}& 0& 0& \frac{\theta {\zeta }_{1}}{\gamma }\\ 0& 0& -\gamma +\frac{\beta \theta }{\gamma }-{\delta }_{1}-{\xi }_{1}& {\eta }_{2}& 0\\ 0& 0& 0& -\gamma -{\delta }_{2}-{\eta }_{2}-{\xi }_{2}& 0\\ 0& 0& \vartheta & 0& -\psi \end{array}\right)$$

This is after acquiring the eigenvalues of ${J}_{{E}_{0}}$, it has the form of,

${\lambda }_{1}=-\gamma , {\lambda }_{2}=-\psi , { \lambda }_{3}=-\gamma -{\eta }_{1} , {\lambda }_{4}=-\gamma +\frac{\beta \theta }{\gamma }-{\delta }_{1}-{\xi }_{1}, {\lambda }_{5}=-\gamma -{\delta }_{2}-{\eta }_{2}-{\xi }_{2}.$As a result, it can be seen that this equilibrium point is stable if $\frac{\beta \theta }{\gamma (\gamma +{\delta }_{1}+{\xi }_{1})}<1$ which corresponds to ${\mathcal{R}}_{0}<1$.

As for the EEP, the Jacobian matrix at ${E}_{1}$ can be found in the form,

$${J}_{{E}_{1}}=\left(\begin{array}{ccccc}\frac{-\beta \theta +\gamma {\delta }_{1}}{\gamma +{\xi }_{1}}& {\eta }_{1}& -\gamma -{\xi }_{1}& {\delta }_{2}& -\frac{{\zeta }_{1}(\gamma +{\delta }_{1}+{\xi }_{1})}{\beta }\\ 0& -\gamma -{\eta }_{1}& 0& 0& \frac{{\zeta }_{1}(\gamma +{\delta }_{1}+{\xi }_{1})}{\beta }\\ -\gamma +\frac{\beta \theta -\gamma {\delta }_{1}}{\gamma +{\xi }_{1}}& 0& 0& {\eta }_{2}& -\frac{{\zeta }_{2}(-\gamma +\frac{\beta \theta -\gamma {\delta }_{1}}{\gamma +{\xi }_{1}})}{\beta }\\ 0& 0& 0& -\gamma -{\delta }_{2}-{\eta }_{2}-{\xi }_{2}& \frac{{\zeta }_{2}(-\gamma +\frac{\beta \theta -\gamma {\delta }_{1}}{\gamma +{\xi }_{1}})}{\beta }\\ 0& 0& \vartheta & 0& -\psi \end{array}\right)$$

The eigenvalues are,

$$\left\{-\gamma -{\eta }_{1},-\gamma -{\eta }_{2}-{\delta }_{2}-{\xi }_{2},-\frac{\psi }{2}-\frac{\sqrt{{\psi }^{2}-4v{\zeta }_{2}\left(\frac{-\gamma +\frac{\beta \theta -\gamma {\delta }_{1}}{\gamma +{\xi }_{1}}}{\beta }\right)}}{2},-\frac{\psi }{2}+\frac{\sqrt{{\psi }^{2}-4v{\zeta }_{2}\left(\frac{-\gamma +\frac{\beta \theta -\gamma {\delta }_{1}}{\gamma +{\xi }_{1}}}{\beta }\right)}}{2},-\beta \left[\frac{\beta \theta -\gamma \left({\delta }_{1}+{\xi }_{1}+\gamma \right)}{\beta \left({\xi }_{1}+\gamma \right)}\right] \right\},$$

This equilibrium point is stable if $\frac{\beta \theta }{\gamma (\gamma +{\delta }_{1}+{\xi }_{1})}<1$. This means that ${\mathcal{R}}_{0}>1$. Similarly, the endemic stability analysis during lockdown can be verified.

Next, we will simulate the results of each of the compartments model (1) through the LADM.

## Proposed technique

Here, we will provide the main steps of applying the LADM for investigating the dynamics of model (1). We first need the following definitions.

### Definition 6.1

Reference^37^ *A function can be defined as an exponentially bounded of order *$\sigma \in R$* if it satisfies the condition that *$\parallel f(t)\parallel \le M{e}^{\sigma t},$* for some real constant* $M>0.$

### Definition 6.2

References^37–39^ *The Caputo fractional derivative can be defined in the form*,

22 $$\mathscr{L}\{{}_{0}^{C}{D}_{t}^{\alpha }f(t)\}={s}^{\sigma }\{f(t)\}-\sum_{k=0}^{m}{s}^{\sigma -k-1}{f}^{(k)}(0),$$

*where *$m=\sigma +1,$* and *$[\alpha ]$* is the integer part of the order *$\sigma$*. Then, we get the following*

23 $$\mathscr{L}({t}^{\sigma })=\frac{\Gamma (\sigma +1)}{{s}^{(\sigma +1)}}, \sigma \in {R}^{+}.$$

These definitions will be used to discuss the general procedure of the proposed technique for simulating the model (1). The first step is to apply the Laplace transform to Eq. (1) which will result

24 $$\left\{\begin{array}{l}\mathscr{L}\left({}_{0}^{C}{D}_{t}^{\alpha }{S}_{1}(t)\right)=\mathscr{L}\left\{\Theta -\beta {S}_{1}(t)I(t)-{\zeta }_{1}{S}_{1}(t)R(t)-\gamma {S}_{1}(t)+{\delta }_{1}I(t)+{\delta }_{2}T(t)+{\eta }_{1}{S}_{2}(t)\right\},\\ \mathscr{L}\left({}_{0}^{C}{D}_{t}^{\alpha }{S}_{2}(t)\right)=\mathscr{L}\left\{{\zeta }_{1}{S}_{1}(t)R(t)-\gamma {S}_{2}(t)-{\eta }_{1}{S}_{2}(t)\right\},\\ \mathscr{L}\left({}_{0}^{C}{D}_{t}^{\alpha }I(t)\right)=\mathscr{L}\left\{\beta {S}_{1}(t)I(t)-{\delta }_{1}I(t)-{\xi }_{1}I(t)-\gamma I(t)-{\zeta }_{2}I(t)R(t)+{\eta }_{2}T(t)\right\},\\\mathscr{L} \left({}_{0}^{C}{D}_{t}^{\alpha }T(t)\right)=\mathscr{L}\left\{{\zeta }_{2}I(t)R(t)-\gamma T(t)-{\eta }_{2}T(t)-{\delta }_{2}T(t)-{\xi }_{2}T(t)\right\},\\ \mathscr{L}\left({}_{0}^{C}{D}_{t}^{\alpha }R(t)\right)=\mathscr{L}\left\{\vartheta I(t)-\psi R(t)\right\}.\end{array}\right.$$

Then, the next step is to apply formula (22) to (24), we conclude,

25 $$\left\{\begin{array}{l}{s}^{\alpha }\mathscr{L}\left({S}_{1}\right)-{s}^{\alpha -1}{S}_{1}\left(0\right)=\frac{\Theta }{s}-\beta\mathscr{L} ({S}_{1}I)-{\zeta }_{1}\mathscr{L}({S}_{1}R)-\gamma {\mathscr{L}(S}_{1})+{\delta }_{1}\mathscr{L}\left(I\right)+{\delta }_{2}\mathscr{L}\left(T\right)+{\eta }_{1}\mathscr{L}{(S}_{2}),\\ {s}^{\alpha }\mathscr{L}\left({S}_{2}\right)-{s}^{\alpha -1}{S}_{2}\left(0\right)={\zeta }_{1}\mathscr{L}({S}_{1}R)-\gamma \mathscr{L}{(S}_{2})-{\eta }_{1}\mathscr{L}{(S}_{2}),\\ {s}^{\alpha }\mathscr{L}\left(I\right)-{s}^{\alpha -1}I\left(0\right)=\beta \mathscr{L}\left({S}_{1}I\right)-{\delta }_{1}\mathscr{L}\left(I\right)-{\xi }_{1}\mathscr{L}\left(I\right)-\gamma \mathscr{L}\left(I\right)-{\zeta }_{2}\mathscr{L}\left(IR\right)+{\eta }_{2}\mathscr{L}\left(T\right),\\ {s}^{\alpha }\mathscr{L}\left(T\right)-{s}^{\alpha -1}T\left(0\right)={\zeta }_{2}\mathscr{L}\left(IR\right)-\gamma \mathscr{L}\left(T\right)-{\eta }_{2}\mathscr{L}\left(T\right)-{\delta }_{2}\mathscr{L}\left(T\right)-{\xi }_{2}\mathscr{L}\left(T\right),\\ {s}^{\alpha }\mathscr{L}\left(R\right)-{s}^{\alpha -1}R\left(0\right)=\vartheta \mathscr{L}\left(I\right)-\psi \mathscr{L}\left(R\right).\end{array}\right.$$

The next step is to apply the initial conditions defined in Eq. (2), then we get,

26 $$\left\{\begin{array}{l}\mathscr{L}\left({S}_{1}\right)=\frac{{H}_{1}}{s}+\frac{\Theta }{{s}^{\alpha +1}}-\frac{\beta }{{s}^{\alpha }}\mathscr{L}({S}_{1}I)-\frac{{\zeta }_{1}}{{s}^{\alpha }}\mathscr{L}({S}_{1}R)-\frac{\gamma }{{s}^{\alpha }}\mathscr{L}{(S}_{1})+\frac{{\delta }_{1}}{{s}^{\alpha }}\mathscr{L}\left(I\right)+\frac{{\delta }_{2}}{{s}^{\alpha }}\mathscr{L}\left(T\right)+{\frac{{\eta }_{1}}{{s}^{\alpha }}\mathscr{L}(S}_{2}),\\ \mathscr{L}\left({S}_{2}\right)=\frac{{H}_{2}}{s}+\frac{{\zeta }_{1}}{{s}^{\alpha }}\mathscr{L}({S}_{1}R)-\frac{\gamma }{{s}^{\alpha }}\mathscr{L}{(S}_{2})-\frac{{\eta }_{1}}{{s}^{\alpha }}\mathscr{L}{(S}_{2}),\\ \mathscr{L}\left(I\right)=\frac{{H}_{3}}{s}+\frac{\beta }{{s}^{\alpha }}\mathscr{L}\left({S}_{1}I\right)-\frac{{\delta }_{1}}{{s}^{\alpha }}\mathscr{L}\left(I\right)-\frac{{\xi }_{1}}{{s}^{\alpha }}\mathscr{L}\left(I\right)-\frac{\gamma }{{s}^{\alpha }}\mathscr{L}\left(I\right)-\frac{{\zeta }_{2}}{{s}^{\alpha }}\mathscr{L}\left(IR\right)+\frac{{\eta }_{2}}{{s}^{\alpha }}\mathscr{L}\left(T\right),\\\mathscr{L} \left(T\right)=\frac{{H}_{4}}{s}+\frac{{\zeta }_{2}}{{s}^{\alpha }}\mathscr{L}\left(IR\right)-\frac{\gamma }{{s}^{\alpha }}\mathscr{L}\left(T\right)-\frac{{\eta }_{2}}{{s}^{\alpha }}\mathscr{L}\left(T\right)-\frac{{\delta }_{2}}{{s}^{\alpha }}\mathscr{L}\left(T\right)-\frac{{\xi }_{2}}{{s}^{\alpha }}\mathscr{L}\left(T\right),\\ \mathscr{L}\left(R\right)=\frac{{H}_{5}}{s}+\frac{\vartheta }{{s}^{\alpha }}\mathscr{L}\left(I\right)-\frac{\psi }{{s}^{\alpha }}\mathscr{L}(R).\end{array}\right.$$

It can be noticed from Eq. (26) that the resulting solution is in the form of an infinite series. Next, let the values of $A=I{S}_{1}, B=IR,$ and $C=R{S}_{1}$ to apply the ADM technique. We assume the following form of solution.

27 $${S}_{1}\left(t\right)=\sum_{n=0}^{\infty }{S}_{1,n}\left(t\right), {S}_{2}\left(t\right)=\sum_{n=0}^{\infty }{S}_{2,n}\left(t\right), T\left(t\right)=\sum_{n=0}^{\infty }{T}_{n}\left(t\right), I\left(t\right)=\sum_{n=0}^{\infty }{I}_{n}\left(t\right), R(t)=\sum_{n=0}^{\infty }{R}_{n}\left(t\right).$$

Next, we treat the nonlinear part of the main model as,

28 $$A=\sum_{n=0}^{\infty }{A}_{n}, B=\sum_{n=0}^{\infty }{B}_{n}, C=\sum_{n=0}^{\infty }{C}_{n} ,$$

Hence, ${A}_{n}, { B}_{n},$ and ${C}_{{\text{n}}}$ can be found with the aid of a convolution procedure as,

29 $$\left\{\begin{array}{c}{A}_{n}=\frac{1}{\Gamma \left(n+1\right)}{\left(\frac{d}{d\varepsilon }\right)}^{n}{\left[\sum_{i=0}^{n}{{\varepsilon }^{i}I}_{i}\sum_{i=0}^{n}{{\varepsilon }^{i}S}_{1,i}\right]}_{\varepsilon =0},\\ {B}_{n}=\frac{1}{\Gamma \left(n+1\right)}{\left(\frac{d}{d\varepsilon }\right)}^{n}{\left[\sum_{i=0}^{n}{{\varepsilon }^{i}I}_{i}\sum_{i=0}^{n}{{\varepsilon }^{i}R}_{i}\right]}_{\varepsilon =0},\\ {C}_{n}=\frac{1}{\Gamma \left(n+1\right)}{\left(\frac{d}{d\varepsilon }\right)}^{n}{\left[\sum_{i=0}^{n}{{\varepsilon }^{i}R}_{i}\sum_{i=0}^{n}{{\varepsilon }^{i}S}_{1,i}\right]}_{\varepsilon =0}.\end{array}\right.$$

Substituting Eq. (27) and Eq. (28) into Eq. (26) and equaling both sides give the following:

$$\mathscr{L}\left({S}_{\mathrm{1,0}}\right)=\frac{{H}_{1}}{s}, \mathscr{L}\left({S}_{\mathrm{2,0}}\right)=\frac{{H}_{2}}{s}, \mathscr{L}\left({I}_{0}\right)=\frac{{H}_{3}}{s}, \mathscr{L}\left({T}_{0}\right)=\frac{{H}_{4}}{s} , \mathscr{L}\left({R}_{0}\right)=\frac{{H}_{5}}{s}$$

$$\left\{\begin{array}{l}\mathscr{L}\left({S}_{\mathrm{1,1}}\right)=\frac{\Theta }{{s}^{\alpha +1}}-\frac{\beta }{{s}^{\alpha }}\mathscr{L}\left({A}_{0}\right)-\frac{{\zeta }_{1}}{{s}^{\alpha }}\mathscr{L}\left({C}_{0}\right)-\frac{\gamma }{{s}^{\alpha }}\mathscr{L}\left({S}_{\mathrm{1,0}}\right)+\frac{{\delta }_{1}}{{s}^{\alpha }}\mathscr{L}\left({I}_{0}\right)+\frac{{\delta }_{2}}{{s}^{\alpha }}\mathscr{L}\left({T}_{0}\right)+\frac{{\eta }_{1}}{{s}^{\alpha }}\mathscr{L}\left({S}_{\mathrm{2,0}}\right),\\ \mathscr{L}\left({S}_{\mathrm{2,1}}\right)=\frac{{\zeta }_{1}}{{s}^{\alpha }}\mathscr{L}({C}_{0})-\frac{\gamma }{{s}^{\alpha }}\mathscr{L}\left({S}_{\mathrm{2,0}}\right)-\frac{{\eta }_{1}}{{s}^{\alpha }}\mathscr{L}\left({S}_{\mathrm{2,0}}\right),\\ \mathscr{L}\left({I}_{1}\right)=\frac{\beta }{{s}^{\alpha }}\mathscr{L}\left({A}_{0}\right)-\frac{{\delta }_{1}}{{s}^{\alpha }}\mathscr{L}\left({I}_{0}\right)-\frac{{\xi }_{1}}{{s}^{\alpha }}\mathscr{L}\left({I}_{0}\right)-\frac{\gamma }{{s}^{\alpha }}\mathscr{L}\left({I}_{0}\right)-\frac{{\zeta }_{2}}{{s}^{\alpha }}\mathscr{L}\left({B}_{0}\right)+\frac{{\eta }_{2}}{{s}^{\alpha }}\mathscr{L}\left({T}_{0}\right),\\ \mathscr{L}\left({T}_{1}\right)=\frac{{\zeta }_{2}}{{s}^{\alpha }}\mathscr{L}\left({B}_{0}\right)-\frac{\gamma }{{s}^{\alpha }}\mathscr{L}\left({T}_{0}\right)-\frac{{\eta }_{2}}{{s}^{\alpha }}\mathscr{L}\left({T}_{0}\right)-\frac{{\delta }_{2}}{{s}^{\alpha }}\mathscr{L}\left({T}_{0}\right)-\frac{{\xi }_{2}}{{s}^{\alpha }}\mathscr{L}\left({T}_{0}\right),\\ \mathscr{L}\left({R}_{1}\right)=\frac{\vartheta }{{s}^{\alpha }}\mathscr{L}\left({I}_{0}\right)-\frac{\psi }{{s}^{\alpha }}\mathscr{L}({R}_{0}).\end{array}\right.$$

$$\vdots$$

30 $$\left\{\begin{array}{l}\mathscr{L}\left({S}_{1,n}\right)=-\frac{\beta }{{s}^{\alpha }}\mathscr{L}\left({A}_{n-1}\right)-\frac{{\zeta }_{1}}{{s}^{\alpha }}\mathscr{L}\left({C}_{n-1}\right)-\frac{\gamma }{{s}^{\alpha }}\mathscr{L}\left({S}_{1,n-1}\right)+\frac{{\delta }_{1}}{{s}^{\alpha }}\mathscr{L}\left({I}_{n-1}\right)+\frac{{\delta }_{2}}{{s}^{\alpha }}\mathscr{L}\left({T}_{n-1}\right)+\frac{{\eta }_{1}}{{s}^{\alpha }}\mathscr{L}\left({S}_{2,n-1}\right),\\ \mathscr{L}\left({S}_{2,n}\right)=\frac{{\zeta }_{1}}{{s}^{\alpha }}\mathscr{L}({C}_{n-1})-\frac{\gamma }{{s}^{\alpha }}\mathscr{L}\left({S}_{2,n-1}\right)-\frac{{\eta }_{1}}{{s}^{\alpha }}\mathscr{L}\left({S}_{2,n-1}\right),\\ \mathscr{L}\left({I}_{n}\right)=\frac{\beta }{{s}^{\alpha }}\mathscr{L}\left({A}_{n-1}\right)-\frac{{\delta }_{1}}{{s}^{\alpha }}\mathscr{L}\left({I}_{n-1}\right)-\frac{{\xi }_{1}}{{s}^{\alpha }}\mathscr{L}\left({I}_{n-1}\right)-\frac{\gamma }{{s}^{\alpha }}\mathscr{L}\left({I}_{n-1}\right)-\frac{{\zeta }_{2}}{{s}^{\alpha }}\mathscr{L}\left({B}_{n-1}\right)+\frac{{\eta }_{2}}{{s}^{\alpha }}\mathscr{L}\left({T}_{n-1}\right),\\ \mathscr{L}\left({T}_{n}\right)=\frac{{\zeta }_{2}}{{s}^{\alpha }}\mathscr{L}\left({B}_{n-1}\right)-\frac{\gamma }{{s}^{\alpha }}\mathscr{L}\left({T}_{n-1}\right)-\frac{{\eta }_{2}}{{s}^{\alpha }}\mathscr{L}\left({T}_{n-1}\right)-\frac{{\delta }_{2}}{{s}^{\alpha }}\mathscr{L}\left({T}_{n-1}\right)-\frac{{\xi }_{2}}{{s}^{\alpha }}\mathscr{L}\left({T}_{n-1}\right),\\ \mathscr{L}\left({R}_{n}\right)=\frac{\vartheta }{{s}^{\alpha }}\mathscr{L}\left({I}_{n-1}\right)-\frac{\psi }{{s}^{\alpha }}\mathscr{L}({R}_{n-1}).\end{array}\right.$$

Then, applying for Eq. (30) the inverse Laplace we reach,

$${S}_{\mathrm{1,0}}\left(t\right)={H}_{1}, {S}_{\mathrm{2,0}}\left(t\right)={H}_{2}, {I}_{0}\left(t\right)={H}_{3}, {T}_{0}\left(t\right)={H}_{4}, {R}_{0}(t)={H}_{5}$$

31 $$\left\{\begin{array}{l}{S}_{\mathrm{1,1}}(t)=\left[\Theta -\beta {A}_{0}-{\zeta }_{1}{C}_{0}-\gamma {H}_{1}+{\delta }_{1}{H}_{3}+{\delta }_{2}{H}_{4}+{\eta }_{1}{H}_{2}\right]\frac{{t}^{\alpha }}{\Gamma \left(\alpha +1\right)},\\ {S}_{\mathrm{2,1}}(t)=\left[{\zeta }_{1}{C}_{0}-\gamma {H}_{2}-{\eta }_{1}{H}_{2}\right]\frac{{t}^{\alpha }}{\Gamma \left(\alpha +1\right)},\\ {I}_{1}(t)=\left[\beta {A}_{0}-{\delta }_{1}{H}_{3}-{\xi }_{1}{H}_{3}-\gamma {H}_{3}-{\zeta }_{2}{B}_{0}+{\eta }_{2}{H}_{4}\right]\frac{{t}^{\alpha }}{\Gamma \left(\alpha +1\right)},\\ {T}_{1}(t)=\left[{\zeta }_{2}{B}_{0}-\gamma {H}_{4}-{\eta }_{2}{H}_{4}-{\delta }_{2}{H}_{4}-{\xi }_{2}{H}_{4}\right]\frac{{t}^{\alpha }}{\Gamma \left(\alpha +1\right)},\\ {R}_{1}(t)=\left[\vartheta {H}_{3}-\psi {H}_{5}\right]\frac{{t}^{\alpha }}{\Gamma \left(\alpha +1\right)}.\end{array}\right.$$

Similarly, at the final step, we get the rest of the terms as infinite series as,

32 $$\left\{\begin{array}{l}{S}_{1}\left(t\right)=\sum_{n=0}^{\infty }{S}_{1,n}\left(t\right)={H}_{1}+\left[\Theta -\beta {A}_{0}-{\zeta }_{1}{C}_{0}-\gamma {H}_{1}+{\delta }_{1}{H}_{3}+{\delta }_{2}{H}_{4}+{\eta }_{1}{H}_{2}\right]\frac{{t}^{\alpha }}{\Gamma \left(\alpha +1\right)}+\cdots ,\\ {S}_{2}(t)=\sum_{n=0}^{\infty }{S}_{2,n}\left(t\right)={H}_{2}+\left[{\zeta }_{1}{C}_{0}-\gamma {H}_{2}-{\eta }_{1}{H}_{2}\right]\frac{{t}^{\alpha }}{\Gamma \left(\alpha +1\right)}+\cdots ,\\ T\left(t\right)=\sum_{n=0}^{\infty }{T}_{n}\left(t\right)={H}_{3}+\left[\beta {A}_{0}-{\delta }_{1}{H}_{3}-{\xi }_{1}{H}_{3}-\gamma {H}_{3}-{\zeta }_{2}{B}_{0}+{\eta }_{2}{H}_{4}\right]\frac{{t}^{\alpha }}{\Gamma \left(\alpha +1\right)}+\cdots ,\\ I\left(t\right)=\sum_{n=0}^{\infty }{I}_{n}\left(t\right)={H}_{4}+\left[{\zeta }_{2}{B}_{0}-\gamma {H}_{4}-{\eta }_{2}{H}_{4}-{\delta }_{2}{H}_{4}-{\xi }_{2}{H}_{4}\right]\frac{{t}^{\alpha }}{\Gamma \left(\alpha +1\right)}+\cdots ,\\ R(t)=\sum_{n=0}^{\infty }{R}_{n}\left(t\right)={H}_{5}+\left[\vartheta {H}_{3}-\psi {H}_{5}\right]\frac{{t}^{\alpha }}{\Gamma \left(\alpha +1\right)}+\cdots .\end{array}\right.$$

Equation (32) solves the main SITR model of Eq. (1) which will be illustrated in the next section.

## Numerical simulations

In this section, we will demonstrate the simulations for model (1) using multiple approaches. First, in subsection “Laplace Adomian decomposition technique”, we will illustrate the results obtained by adapting the Laplace Adomian decomposition technique (LADM) for different values of the fractional order $\alpha$. In addition, in Sect. “Numerical technique”, a numerical verification of the obtained results by the LAMD is presented by the known Adams–Bashforth-Moulton method (ABM). To further validate the obtained results from both techniques, we compare these results from real data from Italy during the lockdown period at the beginning of 2020 where it is witnessed that the obtained results are in good agreement with real data. This proves the effectiveness of the proposed model for simulating the dynamics of the virus.

### Laplace Adomian decomposition technique

**Figure 3:**
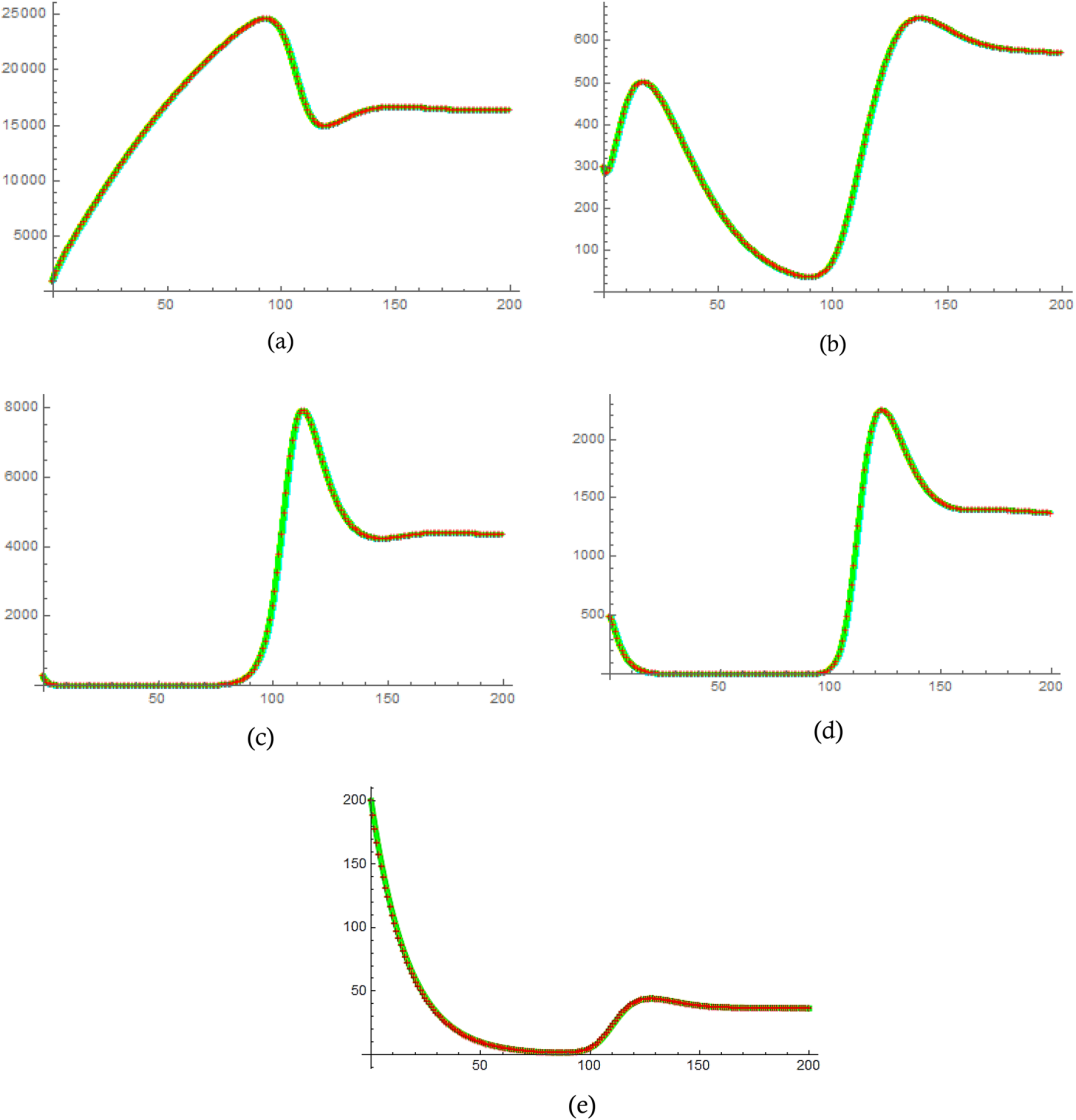
The solution of the copartments (**a**) ${S}_{1}\left(t\right)$, (**b**) ${S}_{2}\left(t\right)$, (**c**) $T(t)$, (**d**) $I(t)$, and (**e**) $R\left(t\right)$ obtained by ODE 45 (REd), LADM (Green) for $\alpha =1,\mathrm{ and }0 < t < 200$.

**Figure 4:**
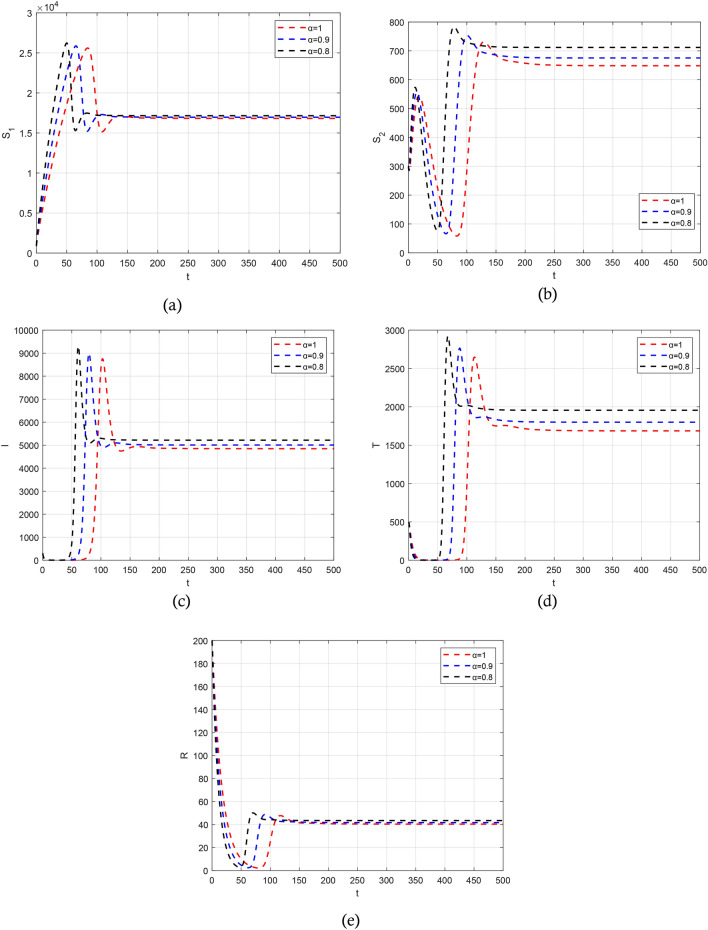
The solution of the copartments (**a**) ${S}_{1}\left(t\right)$, (**b**) ${S}_{2}\left(t\right)$, (**c**) $T(t)$, (**d**) $I(t)$, and (**e**) $R\left(t\right)$ obtained by LADM for different values of $\alpha$.

In this section, we test the effectiveness of the proposed technique by examining the acquired results for model (1) for different $\alpha$. The values of the parameters that have been used for simulating model (1) are summarized in Table 2^36^. The results obtained by ALDM match the exact solutions when $\alpha = 1$. Figure 3 provides a comparison of the results acquired by the LADM and the MATLAB code ODE45 for the different model categories. It is evident from this figure that the proposed technique is efficient and accurate, as it perfectly agrees with the MATLAB code results. The results of the addition of the fractional term can be seen in Fig. 4, where compartments are drawn for varying values of $\alpha =1, 0.9, 0.8, 0.7$. It is evident that the effect of the fractional order is visible as ${S}_{1}$ , $I$ and $T$ compartments decrease gradually while other categories increase at that rate. Furthermore, it can be observed that all categories become more stable for fewer values of $\alpha$ when changing its value, which demonstrates the success of the proposed problem in modeling the COVID-19 pandemic. The charts indicate that an LADM method is an effective tool to better simulate and understand epidemic models with fractional order problems; this is especially noticeable when looking at how they behave at $t=20$ and beyond.

**Table 2:** Values of the main parameters in Eq. (1).

Parameters	Values
${H}_{1}$	900
${H}_{2}$	300
${H}_{3}$	300
${H}_{4}$	497
${H}_{5}$	200
$\Theta$	400
$\beta$	0.000017
${\delta }_{1}$	0.16979
${\delta }_{2}$	0.16979
${\zeta }_{1}$	0.0002
${\zeta }_{2}$	0.002
${\xi }_{1}$	0.03275
${\xi }_{2}$	0.03275
$\gamma$	0.0096
${\eta }_{1}$	0.2
${\eta }_{2}$	0.02
$\nu$	0.0005
$\psi$	0.06

### Numerical technique

In this section, we shall determine the numerical results of model (1) by making use of an effective numerical technique. We employ the Adams–Bashforth-Moulton method, also known as the ABM method, to perform numerical simulations and obtain solutions for the proposed nonlinear fractional order model. The ABM method offers several notable advantages. First and foremost, it enhances the convergence rate of the simulations. One key advantage of the ABM method is its ability to bypass the need for linearization, discretization, and the imposition of physically unrealistic assumptions. By avoiding these limitations, the method provides a more accurate representation of the proposed system. The ABM method is generally stable for a broad class of fractional differential equations (FDEs). Stability is a crucial property in numerical methods since it ensures that the solutions remain bounded and do not exhibit unphysical behavior or divergence. Its efficacy has been demonstrated in solving a wide range of nonlinear FDEs, further emphasizing the suitability of fractional order differential equations for modeling the dynamics of the proposed model realistically. First, we review the fundamentals of the proposed numerical method that has been used to numerically simulate fractional IVPs with Caputo derivatives. The following formulas give a complete presentation of the fractional ABM approach (The same as ${S}_{1}$, all additional states can be discovered).

Suppose that the domain of the solution is [0, *T*] and, $n=\mathrm{0,1},2, \dots ,N,$ where $h=T/N$, ${t}_{n}=nh$. If we assume that ${}_{0}^{C}{D}_{t}^{\alpha }{S}_{1}\left(t\right)={\phi }_{1}\left(t,{S}_{1}\left(t\right),{S}_{2}\left(t\right),I\left(t\right),T\left(t\right),R\left(t\right)\right),$ then,

$${S}_{1}({t}_{n+1})=\sum_{i=0}^{\lceil\alpha \rceil-1}{S}_{\mathrm{1,0}}^{(i)}\frac{{t}_{n+1}^{i}}{i!}+\frac{{h}^{\alpha }}{\Gamma (\alpha +2)}{\phi }_{1}({t}_{n+1},{S}_{1}^{p}({t}_{n+1}))+\frac{{h}^{\alpha }}{\Gamma (\alpha +2)}\sum_{j=0}^{n}{\lambda }_{j,n+1}{\phi }_{1}({t}_{j},{S}_{1}({t}_{j})),$$

$${S}_{1}^{p}({t}_{n+1})=\sum_{i=0}^{\lceil\alpha \rceil-1}{S}_{\mathrm{1,0}}^{(i)}\frac{{t}_{n+1}^{i}}{i!}+\frac{1}{\Gamma (\alpha )}\sum_{k=0}^{n}{\Omega }_{k,n+1}{\phi }_{1}({t}_{k},{S}_{1}({t}_{k})),$$

where

$$\lambda_{j,n + 1} = \left\{ \begin{gathered} n^{\alpha + 1} - (n - \alpha )(n + 1)^{\alpha } ,\,\,\,\,\,\,\,\,\,\,\,\,\,\,\,\,\,\,\,\,\,\,\,\,\,\,\,\,\,\,\,\,\,\,\,\,\,\,\,\,\,\,\,\,\,\,\,{\text{if}}\,\,j = 0, \hfill \\ (n - j + 2)^{\alpha + 1} + (n - j)^{\alpha + 1} - 2(n - j + 1)^{\alpha + 1} ,\,\,\,\,\,{\text{if}}\,\,1 \le j \le n, \hfill \\ 1\,\,\,\,\,\,\,\,\,\,\,\,\,\,\,\,\,\,\,\,\,\,\,\,\,\,\,\,\,\,\,\,\,\,\,\,\,\,\,\,\,\,\,\,\,\,\,\,\,\,\,\,\,\,\,\,\,\,\,\,\,\,\,\,\,\,\,\,\,\,\,\,\,\,\,\,\,\,\,\,\,\,\,\,\,\,\,\,\,\,{\text{if}}\,\,j = n + 1, \hfill \\ \end{gathered} \right.$$

And $$\Omega_{j,n + 1} = \frac{{h^{\alpha } }}{\alpha }((n + 1 - j)^{\alpha } - (n - j)^{\alpha } ).$$

**Figure 5:**
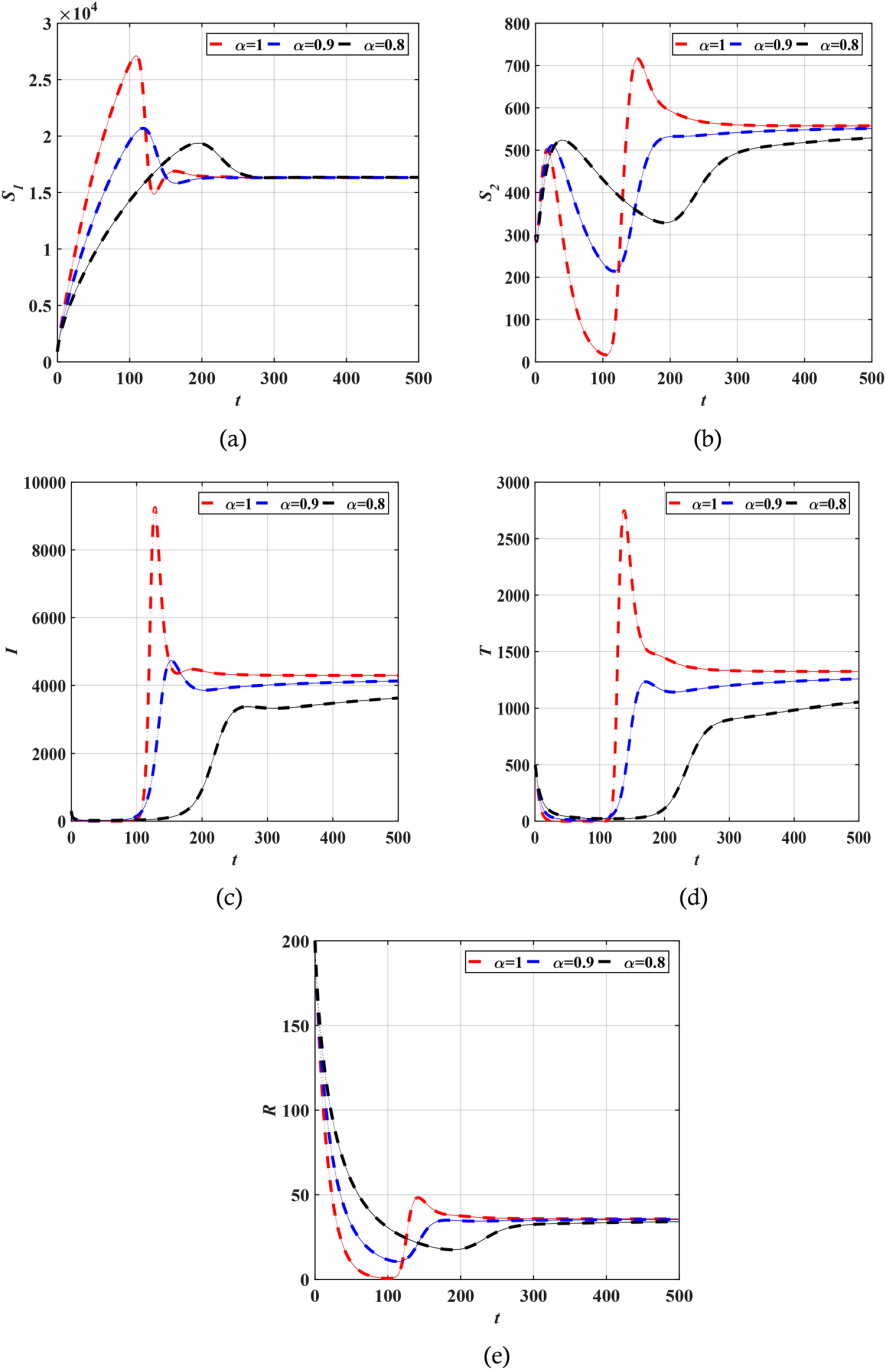
Solution profiles of the different compartments for $\alpha =\mathrm{1,0.9,0.8}$ using the PECE method of ABM.

**Figure 6:**
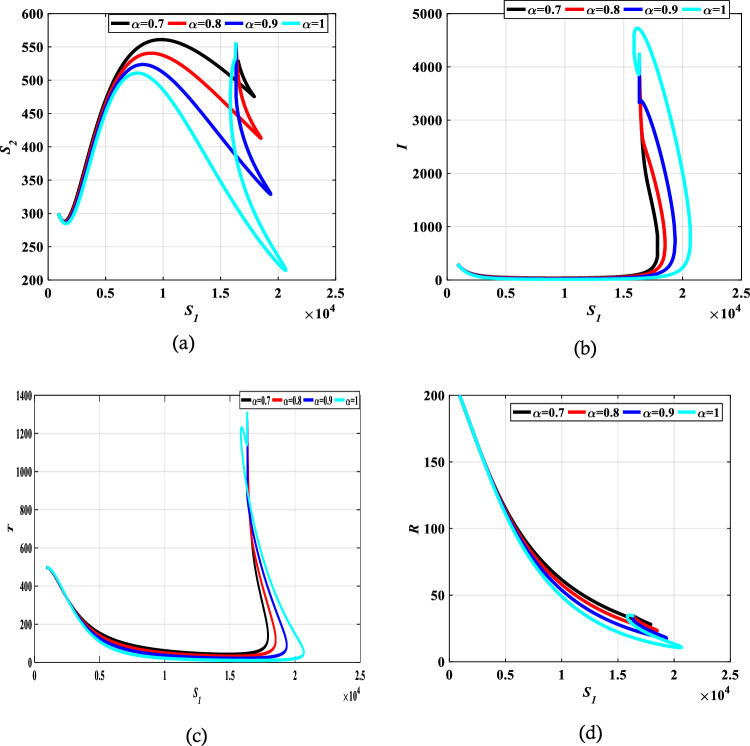
Relation between susceptible ${S}_{1}$ and different comaprtements for $\alpha =0.7, 0.8, 0.9,$ and $\alpha =1$.

**Figure 7:**
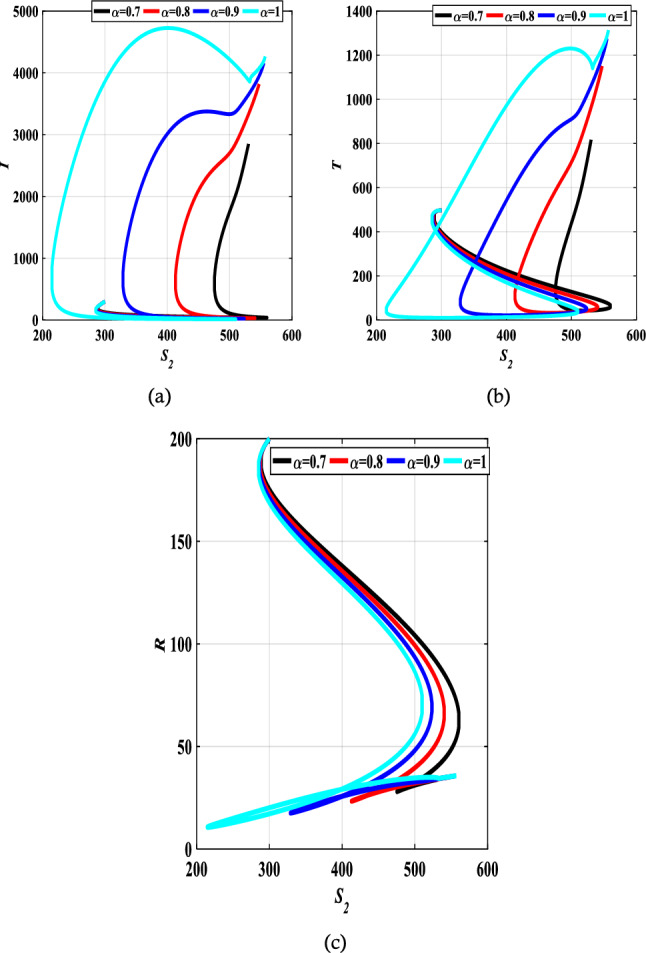
Relation between susceptible ${S}_{2}$ and different comaprtements for $\alpha =0.7, 0.8, 0.9,$ and $\alpha =1$.

**Figure 8:**
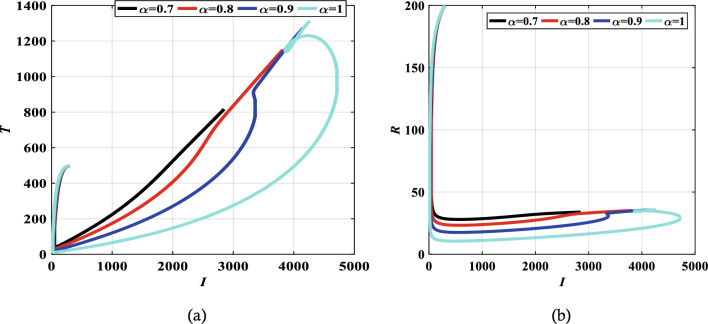
Relation between infected state $I$ and other different compartments for $\alpha =0.7, 0.8, 0.9,$ and $\alpha =1$.

**Figure 9:**
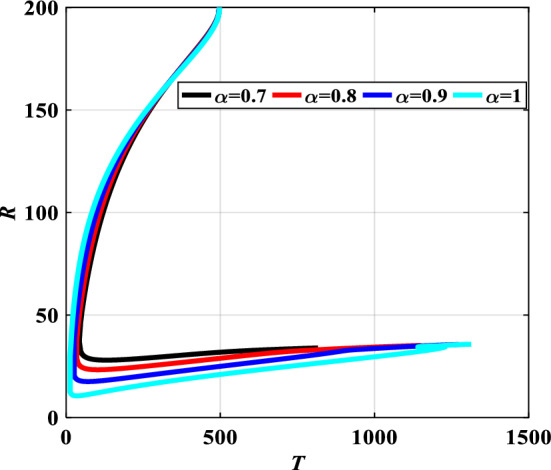
Relation between *T* and *R* for α = 0.7, 0.8, 0.9, and α = 1.

**Figure 10:**
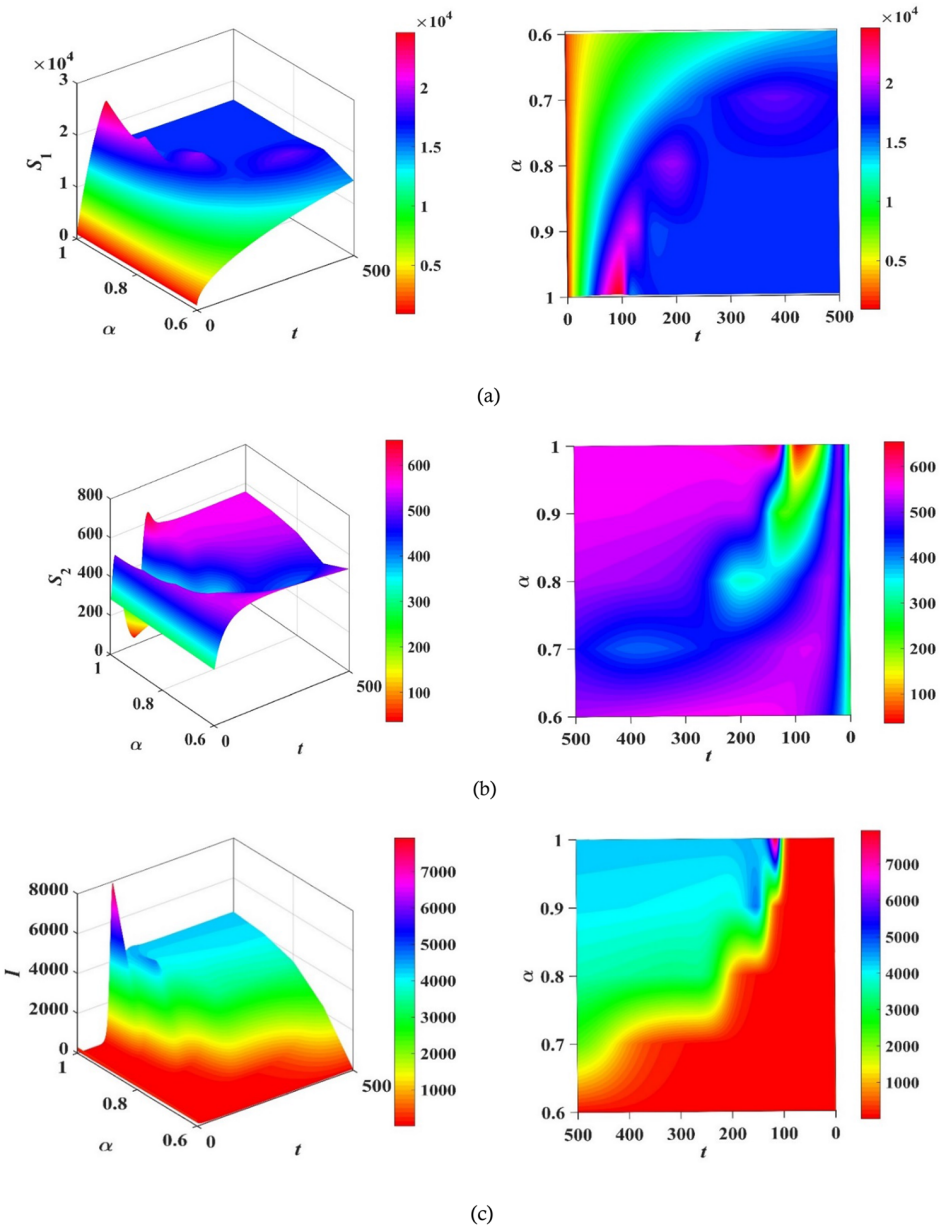

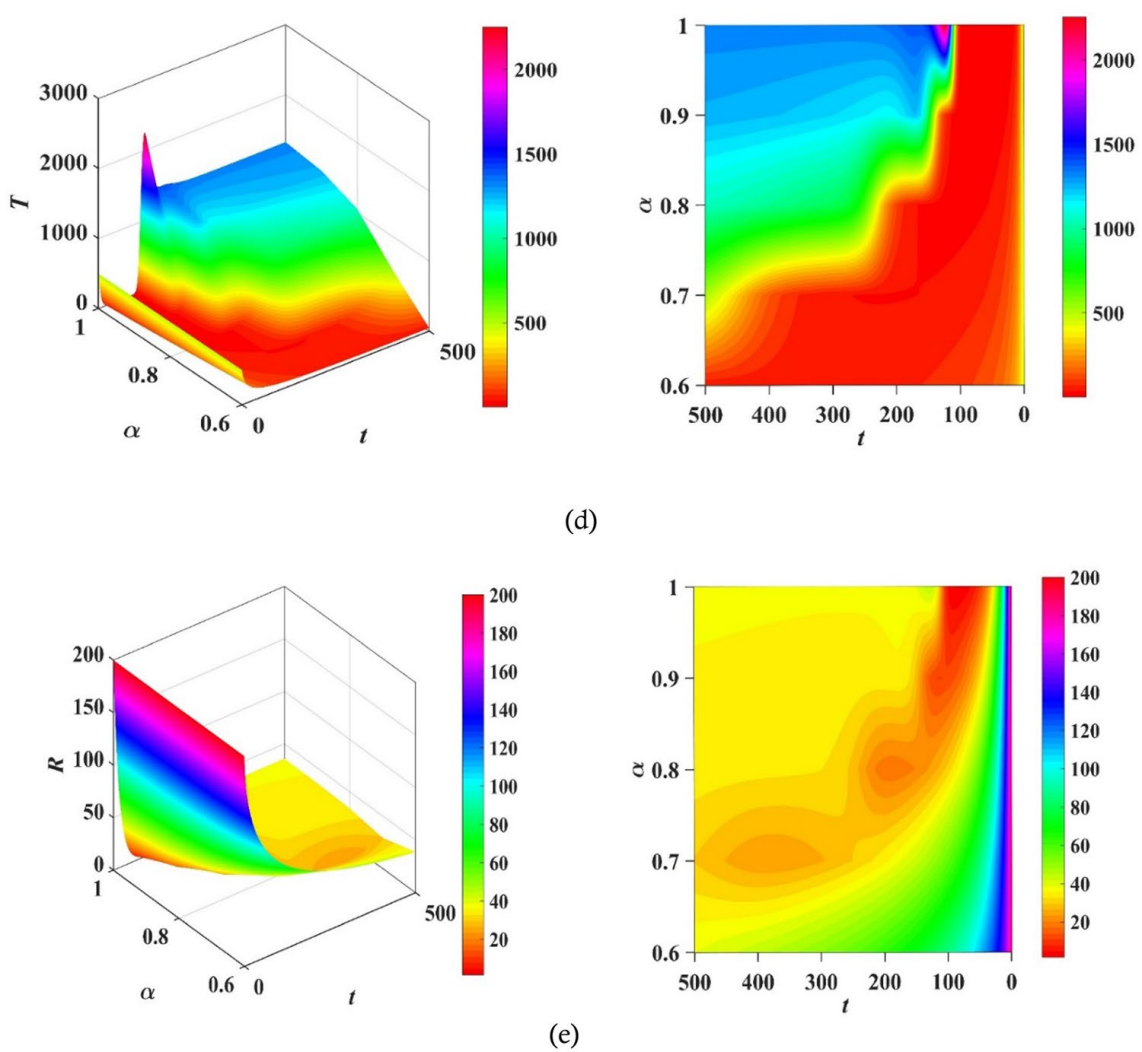
3D plots of the compartment’s populations versus time and fractional order $\alpha$ with the corresponding contour plots.

The authors in Refs.^29,30^ provide details and a complete analysis of the proposed technique. The convergence order of the used fractional Adams–Bashforth-Moulton method is $p={{\text{min}}}(\mathrm{2,1}+\alpha )$, and hence the error is $O({h}^{p})$, see Refs.^43^ for more details. We present the obtained results by the predictor–corrector (PECE) of ABM method for the proposed model. Figure 5 shows the results for simulating the SITR model at $\alpha =0.8, 0.9, 1$. The results are similar to the results obtained by the LADM. Figures 6,7, 8, and 9 depict different phase portraits for the SITR model at $\alpha =0.7, 0.8, 0.9, 1$. Additionally, Fig. 10 displays space plots of different populations of various values of the fractional order $\alpha$. It can be seen from these figures that the obtained results are the same found by the LADM method which proves the effectiveness of both methods for simulating such a model.

### Validation using real data

**Figure 11:**
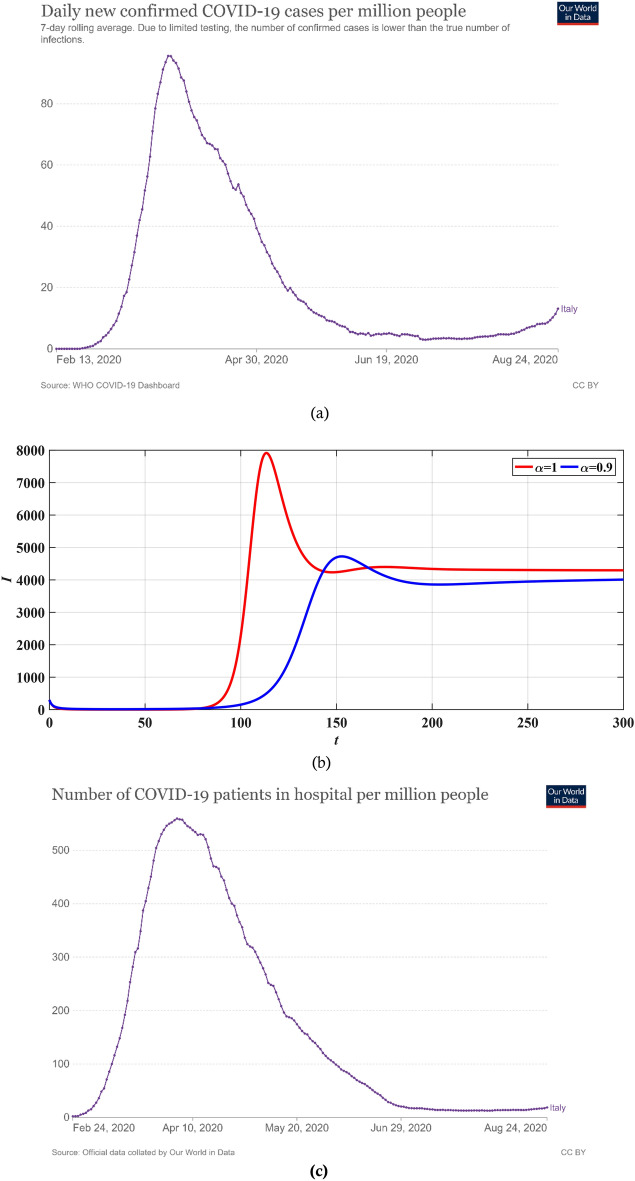

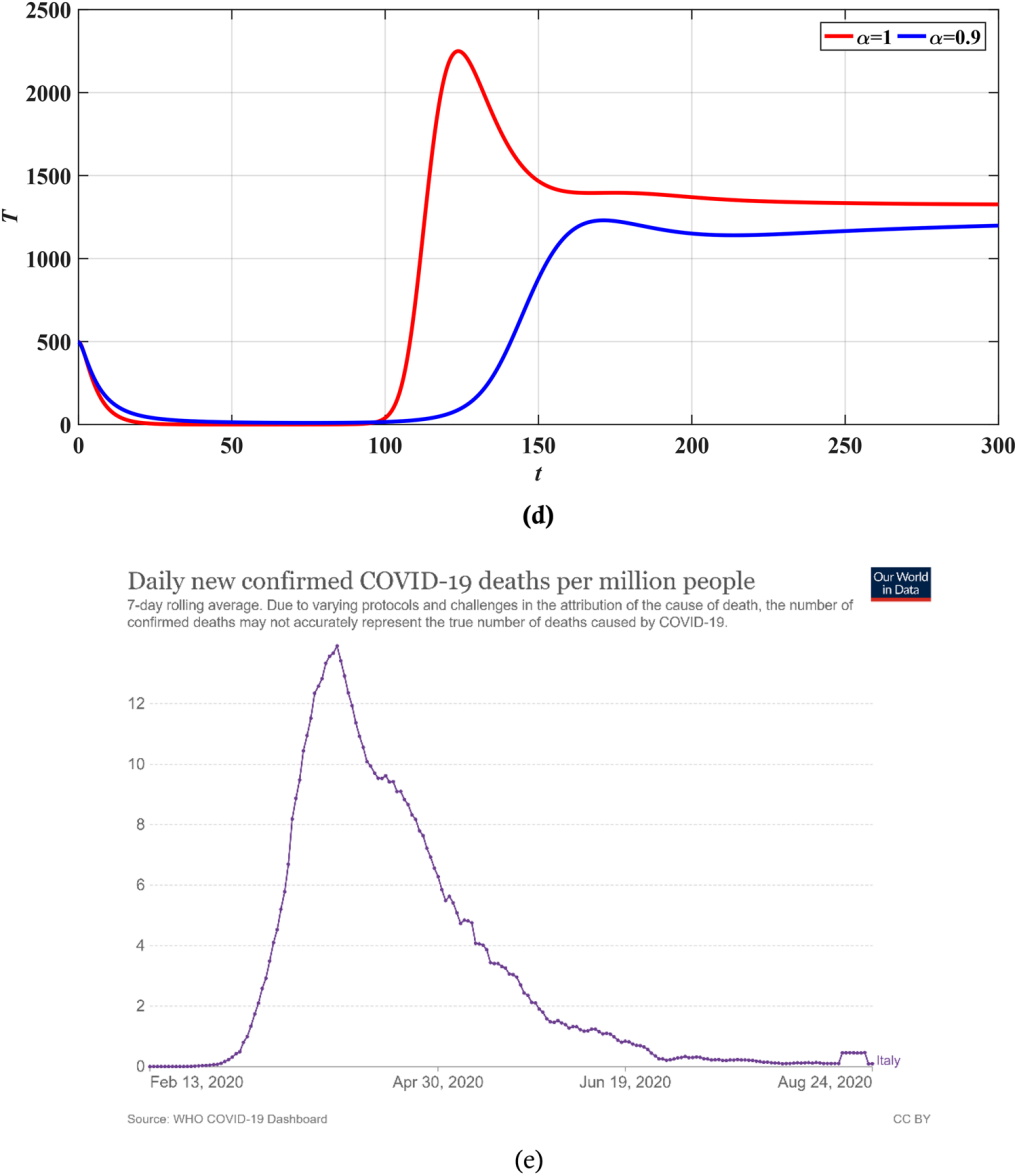
Number of infectd cases from (**a**) real data form Italy^44^, (**b**) form simulation, hospitalized cases from (**c**) real data from Italy^44^, (**d**) form simulation and (**e**) death cases during the lockdown^44^.

In this subsection, we will validate the obtained results from the LADM and numerical techniques by comparing them to real data. We will verify the obtained results with the obtained results from Italy. During the beginning of 2020, especially from March until May 2020, Italy declared the first lockdown for several facilities in the country as a proper reaction to slow done the spread of COVID-19. The number of infected (confirmed) cases per million, number of hospitalized (treated) cases, and number of deaths are shown in Fig. 11. The results reported in Fig. 11 have been collected form World in Data website^44^. In addition, we have run the simulations using the AMB method described in subsection “Numerical technique” over a shorter interval for $0\le x\le 300$. Based on the results of these figures, it can be noticed that during the first lockdown in Italy, after March 2020, the number of infected, hospitalized, and death cases dropped significantly reaching a stabilized behavior until Augst 2020. This is because governments have taken proactive actions such as lockdown, mandate masking and apply social distancing which helps to reduce the spread of the virus. According to Fig. 11, we can notice that a good agreement between the obtained results from the simulation and the real data from Italy is witnessed. For example, Fig. 11a demonstrates the number of infected cases in Italy until August 2020, where it seems that the number of infected cases increased and then began declining as of March 2020 and this can be also seen from Fig. 11b. The adaptation of the fractional order term is noticed in Fig. 11b where changing the value of $\alpha$ from 1 to 0.9 has a great effect on stabilizing the number of infected cases which gives the simulations a better physical understating of the dynamics of the spread of the virus. In addition, the actual number of hospitalized cases in Italy has been graphed in Fig. 11c and compared with the simulated data in (d). The obtained results have a good fit with the real data and reduced the number of hospitalized cases during the lockdown. Finally, the number of deaths in this period is provided in Fig. 11e where the number drops dramatically during the lockdown phase. All of these figures prove that the results from the proposed model fit the real data obtained for Italy.

## Conclusion

This study presents a Caputo fractional SITR model to highlight some new dynamics of the coronavirus COVID-19. The model is composed of four categories: susceptible $S(t)$, infected $I(t)$, treatment $T(t)$, and recovered $R(t)$ at time $t$. Additionally, the susceptible population is further divided into ${S}_{1} (t)$ and ${S}_{2} (t)$ which indicates the susceptible populations that are not under lockdown measurements or lockdown, respectively. To ensure the consistency of the presented model and to obtain the system's equilibrium points, we investigate the boundedness and positivity of the solution. Furthermore, stability analysis is conducted to measure the effect of different values of the parameters. To solve the COVID-19 model, we employ an effective analytical approach known as the Laplace Adomian decomposition method (LADM), which yields accurate results for this problem. To verify the theoretical findings, an efficient LADM technique is employed for multiple of the fractional order $\alpha$. Confinement rules are necessary for managing this pandemic promptly. We can see that is the time of contact between the human populations, all types of compartments become more stable much faster. Therefore, it is clear that wearing mandatory masks and adhering to social distancing are essential for reducing the spread of this pandemic and gaining control over it. To further verify the obtained results, a comparison with real data from Italy was shown during the lockdown. This shows a perfect agreement between the real results and the obtained results of the effect of control measures and lockdown in slowing down the spread of the virus. Thus, we are interested in further exploring this model more thoroughly with more categories taken into account using a similar effective analytic method and comparing it with real data from other countries.

## Data Availability

All data generated or analyzed during this study are included.
